# An electronic nematic liquid in BaNi_2_As_2_

**DOI:** 10.1038/s41467-022-32112-7

**Published:** 2022-08-04

**Authors:** Yi Yao, Roland Willa, Tom Lacmann, Sofia-Michaela Souliou, Mehdi Frachet, Kristin Willa, Michael Merz, Frank Weber, Christoph Meingast, Rolf Heid, Amir-Abbas Haghighirad, Jörg Schmalian, Matthieu Le Tacon

**Affiliations:** 1 Institut für Quantenmaterialien und -technologien, Karlsruher Institut für Technologie, 76021 Karlsruhe, Germany; 2 Institut für Theorie der Kondensierten Materie, Karlsruher Institut für Technologie, 76131 Karlsruhe, Germany; 3 Karlsruhe Nano Micro Facility (KNMFi), Karlsruhe Institute of Technology (KIT), 76344 Eggenstein-Leopoldshafen, Germany

**Keywords:** Electronic properties and materials, Superconducting properties and materials

## Abstract

Understanding the organizing principles of interacting electrons and the emergence of novel electronic phases is a central endeavor of condensed matter physics. Electronic nematicity, in which the discrete rotational symmetry in the electron fluid is broken while the translational one remains unaffected, is a prominent example of such a phase. It has proven ubiquitous in correlated electron systems, and is of prime importance to understand Fe-based superconductors. Here, we find that fluctuations of such broken symmetry are exceptionally strong over an extended temperature range above phase transitions in ${{{{{\rm{Ba}}}}}}{{{{{{\rm{Ni}}}}}}}_{2}{({{{{{{\rm{As}}}}}}}_{1-x}{{{{{{\rm{P}}}}}}}_{x})}_{2}$, the nickel homologue to the Fe-based systems. This lends support to a type of electronic nematicity, dynamical in nature, which exhibits a particularly strong coupling to the underlying crystal lattice. Fluctuations between degenerate nematic configurations cause splitting of phonon lines, without lifting degeneracies nor breaking symmetries, akin to spin liquids in magnetic systems.

## Abstract (web summary)

Electronic nematicity is typically associated with the breaking of rotational symmetry. Here the authors report unusual nematicity in BaNi2As2, manifested in a large splitting of the optical phonon mode above the structural transition temperature, and link it to the coupling between the lattice and nematic fluctuations.

## Introduction

The normal state of unconventional superconductors generally exhibits a variety of exotic electronic states emerging out of the interplay between intertwined orders. It is at least as intriguing as the superconducting state itself. The electronic nematic state is one such exotic state, which has proven particularly insightful in unveiling the properties of Fe-based superconductors. In these materials, nematicity is a canonical example of vestigial order which grows out of the magnetic fluctuations of two degenerate magnetic ground states^1^. It has been proposed that the superconducting pairing is enhanced—even possibly mediated - by quantum critical nematic fluctuations^2^, but their most prominent effect is rooted in their coupling to the crystal lattice. By softening the *C*_66_ shear modulus in e.g. Ba(Fe_1−*x*_Co_*x*_)_2_As_2_, this coupling ultimately yields a lattice distortion at a structural tetragonal-to-orthorhombic phase transition above the superconducting dome^3,4^.

This coupling to the lattice changes the phonon spectra and dispersion, which in turn provides new routes to probe electronic nematicity. In the fluctuating regime, it was recently shown that the spatial dependence of the nematic fluctuations can directly be inferred from the softening of acoustical phonons^5–7^ at small but finite momentum (**q** ≠ **0**). At the Brillouin zone center (**q** = **0**), the largest effects are observed in the ordered phase, through the lifting of the degeneracy of the *a*- and *b*-axis polarized in-plane vibrations of the square FeAs lattice with *E*_g_ symmetry. The resulting relative splitting Δ*ω*/*ω* of the modes in the orthorhombic phase can be as large as 8% in the Fe-based superconductors’ parent compounds such as BaFe_2_As_2_^8–10^ or EuFe_2_As_2_^11^, exceeding by far the expectation based on the small orthorhombicity $\delta=\frac{a-b}{a+b} \sim 1{0}^{-3}$. The much weaker effects reported in non-magnetic FeSe^12^ suggest that the coupling of nematic degrees of freedom to the lattice in Fe-based superconductors primarily occurs through the spin channel rather than through the orbital one^12^.

At room temperature, BaNi_2_As_2_ has a tetragonal crystal structure (space group I4/mmm) similar to BaFe_2_As_2_, but unlike its Fe-counterpart, it is superconducting, albeit below a modest critical temperature *T*_c_ ~ 0.6 K^13^. While earlier electronic structure studies concluded low electronic correlations in this system, pointing at conventional phonon-mediated BCS superconductivity^14,15^, more recent investigations advocate for an exotic normal state, which exhibits a manifold of charge density waves (CDW) instabilities and structural phase transitions interesting in their own right^16–23^, and possible nematic-driven superconducting pairing^24^. No long-range magnetic order has been reported so far, and it has been argued that the CDW plays a role similar to that of magnetism in the Fe-based superconductors^19^, suggesting that BaNi_2_As_2_ could be seen as a charge analogue of BaFe_2_As_2_.

Here we investigate the lattice and electron dynamics of ${{{{{\rm{Ba}}}}}}{{{{{{\rm{Ni}}}}}}}_{2}{({{{{{{\rm{As}}}}}}}_{1-x}{{{{{{\rm{P}}}}}}}_{x})}_{2}$, and report on an exceptionally large splitting of the doubly degenerate Raman active planar vibrations of the NiAs tetraedra. In sharp contrast to the behavior in the iron-based systems, where this splitting was taken as evidence for nematic symmetry breaking, in BaNi_2_(As_1−*x*_)P_x_)_2_ it occurs well above any reported structural phase transition temperatures. This calls for a distinction between the lifting of a degeneracy and a dynamical spectral splitting. We show that our observation can be accounted for by a particularly strong coupling of electronic *B*_1g_ nematic fluctuations, likely of orbital nature^20^, to the lattice degrees of freedom in this material. This indicates that the tetragonal phase of BaNi_2_As_2_ hosts an electronic nematic phase, dynamical in nature. We show that the broadening and splitting of the planar phonons can be described in terms of an entangled superposition of the two degenerate Ising-nematic states that are coupled to a cloud of vibrational quanta. This bears analogies with the phenomenology of spin liquids—dynamical states without long-range magnetic order but long-range entanglement—and suggests in turn that similarly rich physics could be expected in such nematic liquids.

## Results

**Fig. 1:**
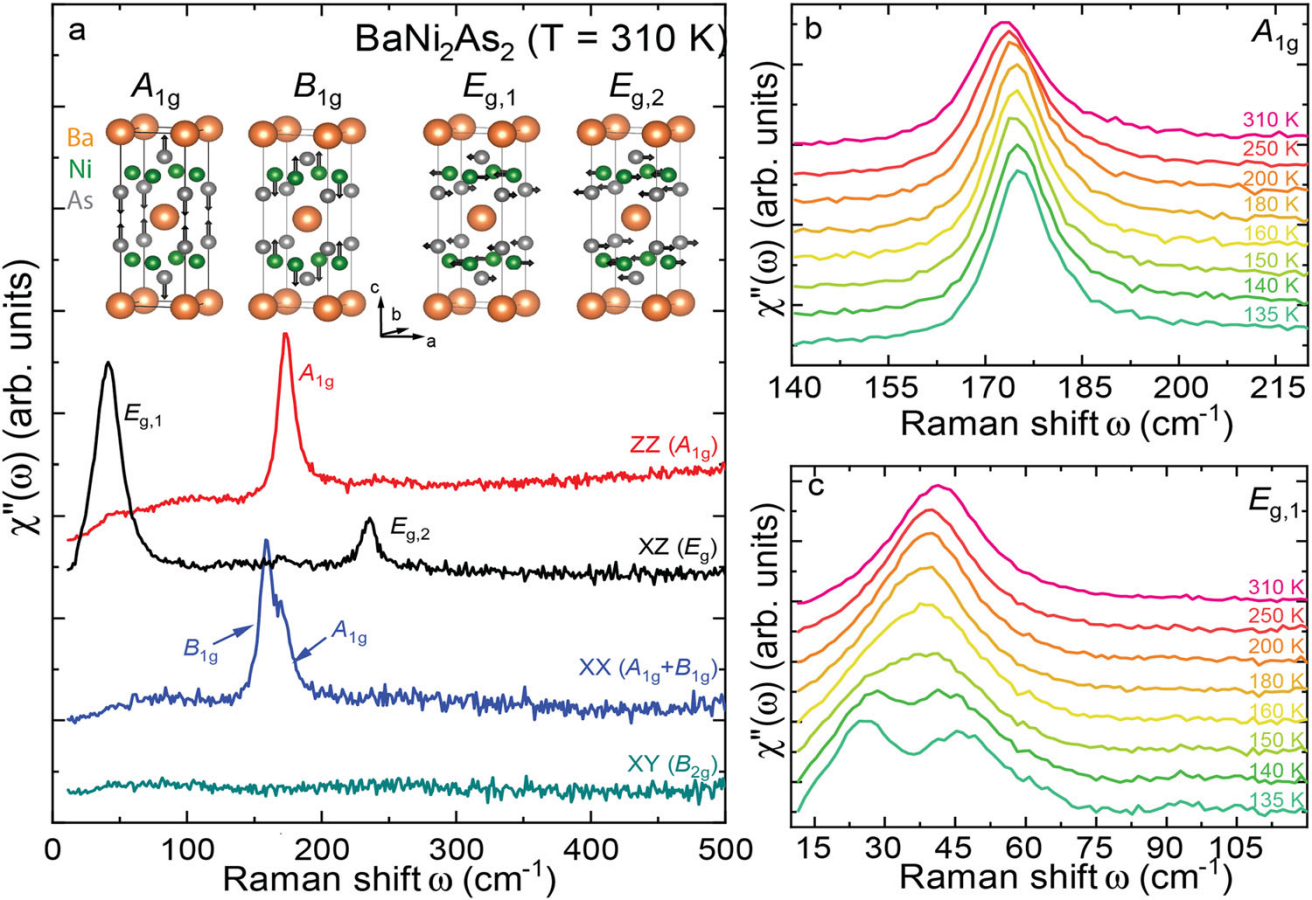
Raman scattering from BaNi_2_As_2_. **a** Raman active phonons of BaNi_2_As_2_, and room temperature Raman spectra obtained in the different incoming and scattered photon polarizations. Detailed view of the temperature dependencies of the *A*_1g_ (**b**) and *E*_g,1_ (**c**) phonons above *T*_Tri_ in BaNi_2_As_2_.

From a point group analysis follows that the tetragonal phase of BaNi_2_As_2_hosts four Raman-active optical phonons of *A*_1g_, *B*_1g_, and *E*_g_ symmetry at the Brillouin zone center. The corresponding eigen-displacements are shown in Fig. 1a. In this figure, we further report on room temperature Raman scattering measurements performed on BaNi_2_As_2_ single crystals. The experiments were carried out in backscattering geometry with *XZ*, *ZZ*, *XX* and *XY* configurations, where the first (respectively second) letter refers to the orientation of the incident (resp. scattered) light polarization with respect to the axis of the tetragonal unit cell (Supplementary Note 3). All four Raman active optical phonon modes were detected. The *A*_1g_ mode is seen in the *ZZ* configuration at 172.9 cm^−1^, as well as in the *XX* channel, where it partially overlaps with the *B*_1g_ mode, at 158.6 cm^−1^. The two modes observed in the *XZ* channel are the doubly degenerate *E*_g_ modes referred to as *E*_g,1_ (41.4 cm^−1^) and *E*_g,2_ (235.2 cm^−1^). With the notable exception of the lowest *E*_g,1_ mode, these energies are in good agreement with the predictions of ab initio calculations (see Supplementary Note 3). These calculations also allowed us to estimate the strength of the electron–phonon coupling for the different modes and revealed that the phonon exhibiting the largest coupling is the *A*_1g_ mode, which consistently displays a weak Fano asymmetry. On the other hand, despite the rather modest calculated electron–phonon coupling, the *E*_g,1_ mode is very broad (full-width-at-half-maximum (FWHM) ~ 22 cm^−1^) at room temperature, indicating additional decay channels.

**Fig. 2:**
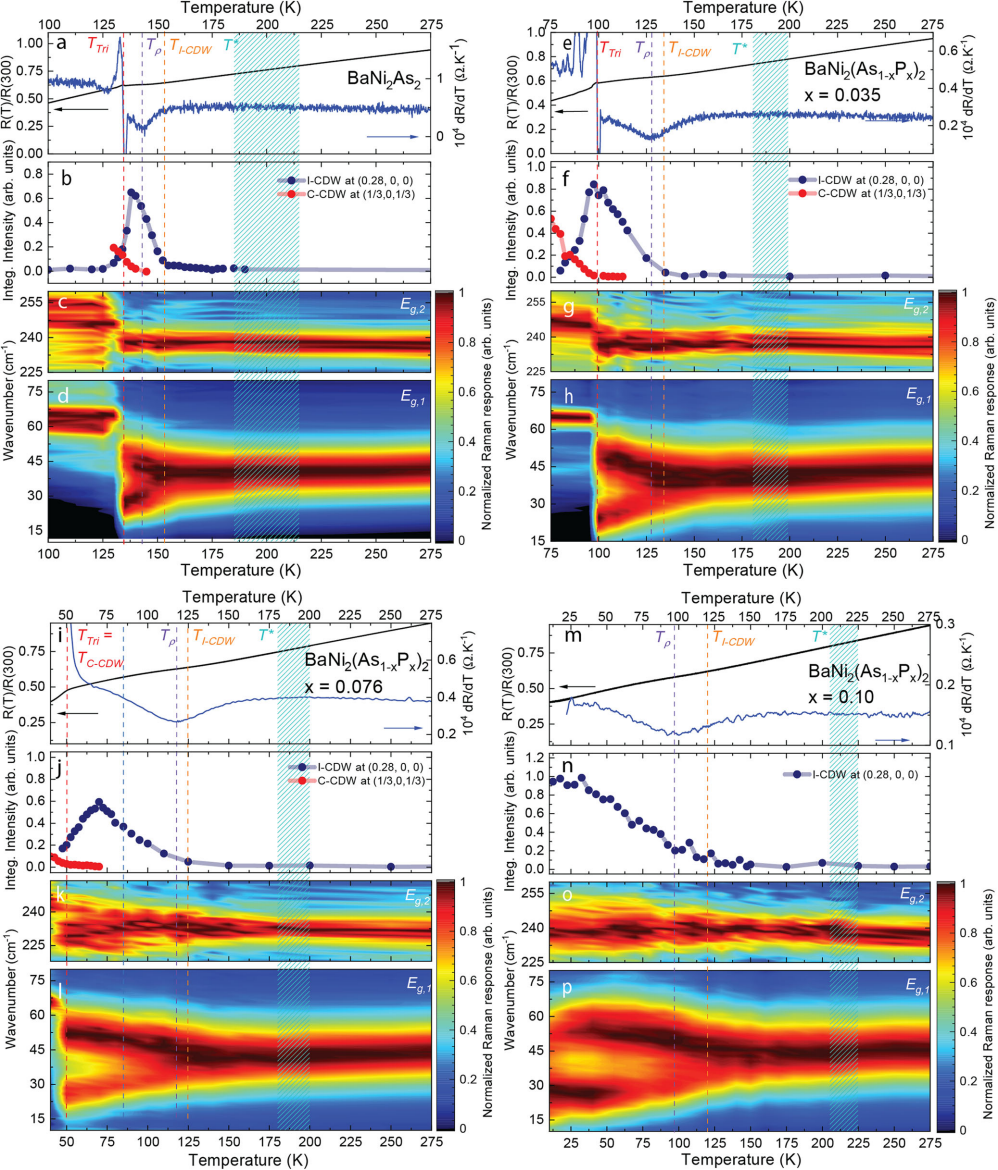
Doping dependence. **a** temperature dependence of the resistivity and its derivative measured upon cooling in BaNi_2_As_2_, **b** of the integrated intensity of the *q*_I-CDW_ = (0.28,0,0) and *q*_C-CDW_ = (1/3,0,1/3) superstructure peaks (measured upon cooling) in BaNi_2_As_2_ (**c**) temperature dependence of the *E*_g,2_ phonon intensity in BaNi_2_As_2_ (after background subtraction and Bose-factor correction, Supplementary Note 3) (**d**) temperature dependence of the *E*_g,1_ phonon intensity in BaNi_2_As_2_ (after background subtraction and bose correction, Supplementary Note 3). **e**, **f**, **g**, **h** same as (**a**, **b**, **c**, **d**) for ${{{{{\rm{Ba}}}}}}{{{{{{\rm{Ni}}}}}}}_{2}{({{{{{{\rm{As}}}}}}}_{1-x}{{{{{{\rm{P}}}}}}}_{x})}_{2}$(*x* = 3.5%) (**i**, **j**, **k**, **l**) same as (**a**, **b**, **c**, **d**) for ${{{{{\rm{Ba}}}}}}{{{{{{\rm{Ni}}}}}}}_{2}{({{{{{{\rm{As}}}}}}}_{1-x}{{{{{{\rm{P}}}}}}}_{x})}_{2}$(*x* =7.6%) (**m**, **n**, **o**, **p**) same as (**a**, **b**, **c**, **d**) for ${{{{{\rm{Ba}}}}}}{{{{{{\rm{Ni}}}}}}}_{2}{({{{{{{\rm{As}}}}}}}_{1-x}{{{{{{\rm{P}}}}}}}_{x})}_{2}$(*x* = 10%). In each panel we have indicated the position of the triclinic transition (upon cooling) *T*_Tri_, of the local minimum of d*R*/d*T* *T*_rho_ and of the temperature at which the I-CDW intensity starts to grow *T*_I-CDW_. The shaded area corresponds to the uncertainty on the determination of T^*^ at which the *E*_g_ phonon starts to broaden.

The singular behavior of the *E*_g,1_ phonon is confirmed upon cooling. The conventional behavior of phonon is exhibited by the *A*_1g_(Fig. 1b) mode which harden and narrow at low temperatures. In contrast, the *E*_g,1_ mode initially softens upon cooling, starts broadening around T^*^ ~ 200 K before splitting at lower temperatures, where two peaks can be resolved; see Fig. 1c and Supplementary Note 3. Just above the first-order transition^13^ to a triclinic phase at *T*_Tri_ = 133 K (on cooling), within which the phonon spectra qualitatively change (Supplementary Note 3 and Fig. 2), the splitting is as large as 22 cm^−1^, that is, more than 50% of the mode’s original frequency.

The degeneracy of an *E*_g_ phonon can only be lifted if the four-fold symmetry of the Ni planes is broken. This occurs across the tetragonal-to-orthorhombic structural transition in the Fe-based compounds Ba(Fe_1−*x*_Co_*x*_)_2_As_2_^8–10^, EuFe_2_As_2_^11^ or FeSe^12^, where *E*_g_ modes split into *B*_2*g*_ and *B*_3*g*_ modes. The largest reported splitting in *A*Fe_2_As_2_ (*A* = Ba or Eu) is ~10 cm^−1^ (~8% of the mode frequency)^8–11^, significantly larger than in FeSe (~2.6 cm^−1^)^12^. In both cases, this splitting is already considered unusually large, in the sense that it exceeds the expectation based on the lattice distortion. The splitting in our measurements is quantitatively much larger and moreover onsets (with a broadening of the mode) at a temperature significantly higher than that at which the four-fold symmetry breaking takes place.

Before discussing the doping dependence of the effect in BaNi_2_As_2_, we briefly review the potential sources of symmetry breaking that could yield a splitting of the *E*_g,1_ phonon. As FeSe, BaNi_2_As_2_does not exhibit any magnetic order but a unidirectional, biaxial, incommensurate CDW (I-CDW) above *T*_Tri_ has recently been reported^16,19,20^. We performed a detailed temperature-dependent x-ray diffraction (XRD) investigation of the intensity of the CDW satellite at *q*_I-CDW_ = (±0.28, 0, 0) (Fig. 2a, note that throughout the manuscript, we will only refer to reciprocal lattice vectors in the tetragonal unit cell). A very weak diffuse scattering signal can be tracked up to room temperature, but a strong increase of the peak intensity is only observed below ~155K. A second-order phase transition consisting of an orthorhombic distortion of the lattice can be detected through high-resolution dilatometry^20^ at ~142 K. In the parent compound, it also manifests itself as a minimum in the derivative of the resistance against temperature d*R*/d*T*, labeled *T*_*ρ*_ (Supplementary Note 2) in Fig. 2a. In contrast to more pronounced distortions, the identification of the twin structure associated with the structural change was limited in our XRD measurements to a broadening of high order Bragg reflections (e.g. (8,0,0)). This turns to our advantage as it allows us to put an upper bound on the corresponding lattice distortion $\delta=\frac{a-b}{a+b} \sim 1{0}^{-4}$, an order of magnitude weaker than that reported in BaFe_2_As_2_ and in good agreement with thermodynamic measurements^21^.

Cooling further, the I-CDW superstructure peak is suppressed in the triclinic phase in which a commensurate CDW (C-CDW) signal develops at *q*_C-CDW_ = (±1/3, 0, ±1/3). We did not detect any additional phase transition in the temperature range at which the *E*_g,1_ mode splitting onsets, and can already conclude at this stage that this splitting is occurring in the tetragonal I4/mmm phase. Our first-principle calculations confirmed that the amplitude of the orthorhombic structural distortion is in all cases much too small to account for the gigantic energy splitting of the *E*_g,1_phonons reported here (Supplementary Note 3). Furthermore, in stark contrast to Fe-based materials^8,12^, the *E*_g,1_ mode splitting increases linearly and does not show any sign of saturation down to *T*_Tri_. In the same temperature range, a subtle broadening of the *E*_g,2_ mode (of much lower intensity) occurs.

**Fig. 3:**
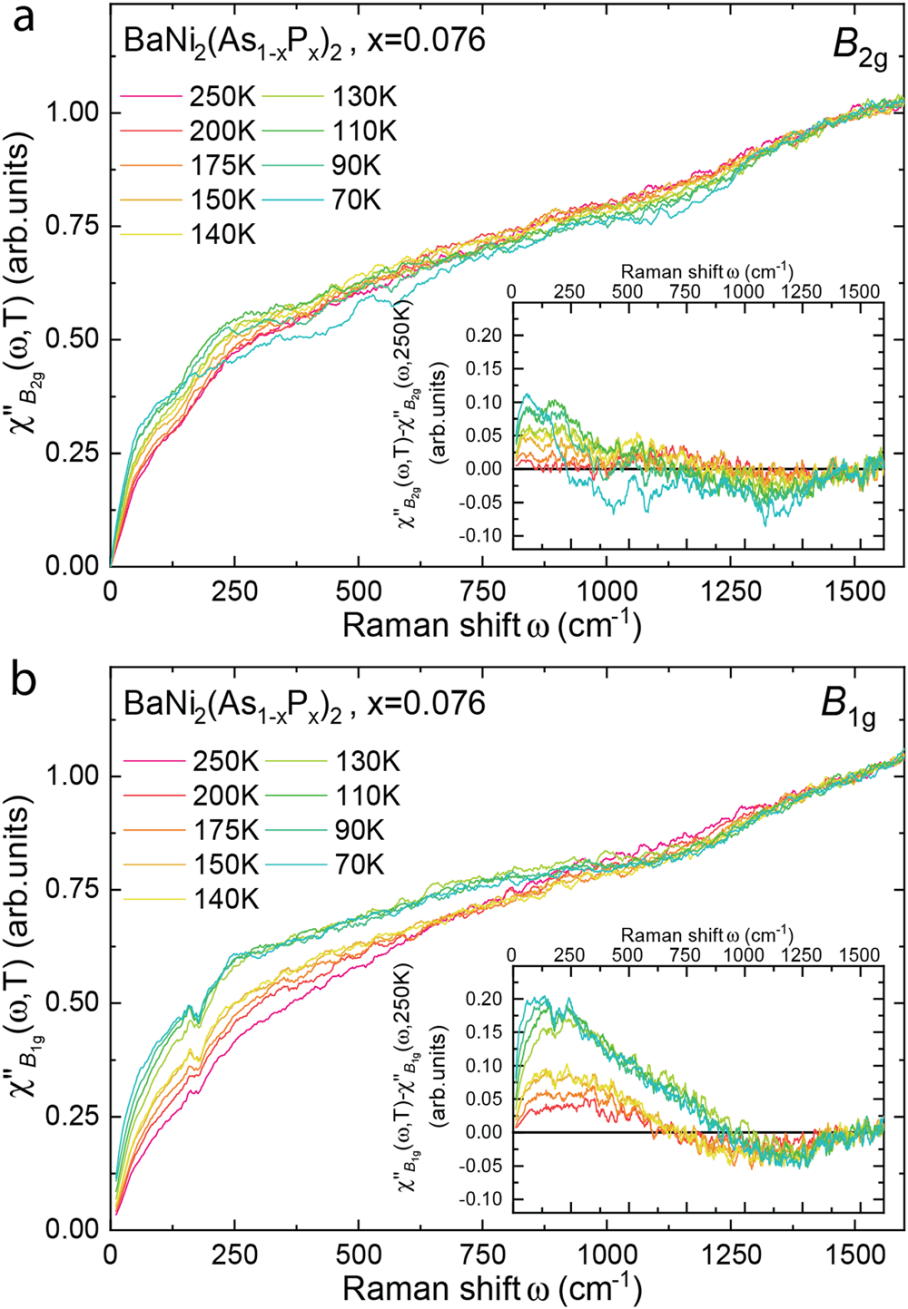
Electronic Raman scattering. **a** *B*_2g_ electronic Raman response ${\chi }_{{B}_{{{{{{\rm{2g}}}}}}}}^{^{\prime\prime} }(\omega,T)$ of ${{{{{\rm{Ba}}}}}}{{{{{{\rm{Ni}}}}}}}_{2}{({{{{{{\rm{As}}}}}}}_{1-x}{{{{{{\rm{P}}}}}}}_{x})}_{2}$(*x* = 7.6%) as a function of temperature. The inset shows the same data after subtraction of the high-temperature response ${\chi }_{{B}_{{{{{{\rm{2g}}}}}}}}^{^{\prime\prime} }(\omega,T)$ - ${\chi }_{{B}_{{{{{{\rm{2g}}}}}}}}^{^{\prime\prime} }(\omega,T=250\,{{{\rm{K}}}})$. **b** *B*_1g_ electronic Raman response ${\chi }_{{B}_{{{{{{\rm{1g}}}}}}}}^{^{\prime\prime} }(\omega,T)$ of ${{{{{\rm{Ba}}}}}}{{{{{{\rm{Ni}}}}}}}_{2}{({{{{{{\rm{As}}}}}}}_{1-x}{{{{{{\rm{P}}}}}}}_{x})}_{2}$(x=7.6%) as a function of temperature. The inset shows the same data after subtraction of the high-temperature response ${\chi }_{{B}_{{{{{{\rm{1g}}}}}}}}^{^{\prime\prime} }(\omega,T)$ - ${\chi }_{{B}_{{{{{{\rm{1g}}}}}}}}^{^{\prime\prime} }(\omega,T=250\,{{{\rm{K}}}})$.

Next, we confirmed the behavior of the *E*_g_ modes by studying the impact of arsenic substitution with phosphorus. This has previously been reported to suppress the triclinic transition^21,25^, and to enhance the orthorhombic distortion^20,21^. In Fig. 2, we show the results for ${{{{{\rm{Ba}}}}}}{{{{{{\rm{Ni}}}}}}}_{2}{({{{{{{\rm{As}}}}}}}_{1-x}{{{{{{\rm{P}}}}}}}_{x})}_{2}$ with *x* = 3.5% (*T*_Tri_ = 95 K) and *x* = 7.6% (*T*_Tri_ = 55 K), for which we observe a similar splitting of the *E*_g,1_ mode, which increases linearly as temperature decreases reaching almost 30 cm^−1^ at *T*_Tri_ (~65% of the mode frequency). Upon further increase of the P-concentration (*x* = 10%), for which the triclinic (and therefore the C-CDW) transition is completely suppressed, the maximal amplitude of the splitting is reduced and appears to saturate at the lowest temperatures. In all doped samples, the strong increase of the I-CDW satellite intensity at *q*_I-CDW_ is smoother than in the parent compound and occurs at a temperature very close to *T*_*ρ*_. The observation of the *E*_g_ phonon broadening and splitting above any lowering of the symmetry of the compound, strongly suggests a coupling of the mode to fluctuations. Given the lack of magnetism in BaNi_2_As_2_, orbital degrees of freedom are the most likely candidates. This can directly be tested using electronic Raman scattering, which is a particularly sensitive probe of the charge fluctuations. In Fig. 3, we show the temperature dependence of the electronic Raman response in the *B*_1g_ and *B*_2g_ channels for the *x* = 6.5% sample (for clarity, the Raman active phonon has been subtracted from the *B*_1g_ spectrum—Supplementary Note 3). In both channels, the electronic response consists of a broad continuum, extending up to 1500 cm^−1^, akin to the particle-hole excitations seen in many correlated metals^26–30^. It apparently displays a conventional metallic behavior, with a smooth increase of the low frequency (≤250 cm^−1^) response upon cooling, reflecting the decrease of the quasiparticle scattering rate Γ (which is inversely proportional to the slope of the Raman response, *χ*″(*ω*)/*ω*∣_*ω*→0_ in the static limit). The main difference between the two channels is quantitative: the low energy *B*_1g_ intensity gain spans over a broader energy range (≥500 cm^−1^) than the *B*_2g_, it is overall larger and accelerates significantly at temperatures where the *E*_g,1_ phonon splitting becomes evident, below ~140 K). This observation is in line with recent elastoresistivity measurements^16,17^ and can be interpreted as a signature of *B*_1g_ nematic fluctuations in BaNi_2_As_2_. In contrast to the electronic nematicity of the Fe-based superconductors, observed in the *B*_2g_ channel, the respective *B*_1g_ response in BaNi_2_As_2_ appears overdamped, suggesting a strong coupling of the lattice to the nematic fluctuations.

Next, we develop a qualitative understanding of the ’splitting’ of the *E*_g_ phonon spectrum, in the absence of a structural phase transition. While the latter would lift the degeneracy and naturally cause such a splitting, we show how when it does not occur, the coupling of nematic fluctuations to the lattice can yield a spectral splitting of a doubly degenerated mode that bears striking similarities to our experimental observation.

We first focus on three phonon branches, a transverse acoustic (TA) mode that couples to nematic order and the two degenerate optical E_*g*_ modes with lattice Hamiltonian ${H}_{{{{{{\rm{latt}}}}}}}=\frac{1}{2}{\sum }_{{{{{{\boldsymbol{q}}}}}}\kappa }{\omega }_{{{{{{\boldsymbol{q}}}}}}\kappa }{a}_{{{{{{\boldsymbol{q}}}}}}\kappa }^{{{\dagger}} }{a}_{{{{{{\boldsymbol{q}}}}}}\kappa }$. Here, *κ* refers to the phonon branch of frequency *ω*_***q****κ*_. Since nematic fluctuations take place at small momenta, we ignore the momentum dependence of the optical modes ${\omega }_{{{{{{\boldsymbol{q}}}}}},{E}_{g}}\approx {\omega }_{0}$. The TA mode frequency ${\omega }_{{{{{{\boldsymbol{q}}}}}},{{{{{\rm{TA}}}}}}}={c}_{{{{\hat{{{\boldsymbol{q}}}}}}}}q$ possesses a direction-dependent sound velocity ${c}_{{{{\hat{{{\boldsymbol{q}}}}}}}}$. For momenta $\hat{q}$ along [100], [010], ${c}_{{{{\hat{{{\boldsymbol{q}}}}}}}}^{2}$ is proportional to the *B*_1g_elastic constant ${C}_{{{{{{{\rm{B}}}}}}}_{1g}}={C}_{11}-{C}_{12}$. In our consideration, we include electronic degrees of freedom with nematic character. Having in mind the absence of magnetic order in this system as well as recent observation of orbital fluctuations above the triclinic transition^20^, suggest that orbital degrees of freedom are the most relevant here. A natural electronic object of proper symmetry is the orbital polarization ${O}_{i}={\sum }_{\sigma=\{\uparrow,\downarrow \}}\left({d}_{i\sigma,xz}^{{{\dagger}} }{d}_{i\sigma,xz}-{d}_{i\sigma,yz}^{{{\dagger}} }{d}_{i\sigma,yz}\right)$ at lattice site *i*; or its Fourier transform in momentum space *O*_***q***_. The Ni 3*d*_*xz*_ and 3*d*_*yz*_ orbitals are symmetry related in the tetragonal phase and broken nematic symmetry implies $\left\langle {O}_{{{{{{\boldsymbol{q}}}}}}}\right\rangle=\left\langle O\right\rangle {\delta }_{{{{{{\boldsymbol{q}}}}}},{{{{{\boldsymbol{0}}}}}}}\,\ne\, 0$. In the following, we draw general conclusions that do not rely on the microscopic origin of such ordering (these go beyond the scope of this paper and will be developed in a subsequent work), nor of its microscopic nature providing that its symmetry allows a coupling to the relevant phonons.

The most direct coupling of the orbital polarization *O*_***q***_ to elastic modes occurs through TA phonons with displacements *u*_*x*,*y*_

1 $${H}_{c,{{{{{\rm{TA}}}}}}}=-\frac{{g}_{{{{{{\rm{TA}}}}}}}}{2i}\mathop{\sum}\limits_{{{{{{\boldsymbol{q}}}}}}}{O}_{{{{{{\boldsymbol{q}}}}}}}({q}_{x}{u}_{-{{{{{\boldsymbol{q}}}}}},x}-{q}_{y}{u}_{-{{{{{\boldsymbol{q}}}}}},y}).$$

This interaction imposes that orbital ordering and the tetragonal-to-orthorhombic transition occur simultaneously. Such coupling between a degenerate electronic state to TA phonons can in principle cause nematic order, akin to a cooperative Jahn–Teller effect^31^. Even when the nematicity is primarily of electronic origin, the nemato-elastic coupling of Eq. (1) will always increase the tendency towards nematic order^32^, consistent with the Jahn-Teller argument. It leads in any event to a lattice softening near the transition and allows probing the nematic susceptibility via measurements of the elastic constant ${C}_{{B}_{1g}}$^3,32^, the elastoresistivity^17^ or the *B*_1g_-Raman response^33^.

By symmetry, the coupling of the orbital polarization to optical E_*g*_ phonon modes is given by

2 $${H}_{c,{E}_{g}}=\frac{{g}_{{E}_{g}}}{2N}\mathop{\sum}\limits_{{{{{{\boldsymbol{k}}}}}},{{{{{\boldsymbol{q}}}}}}}{O}_{{{{{{\boldsymbol{q}}}}}}}({u}_{{{{{{\boldsymbol{k}}}}}}x}{u}_{{{{{{\boldsymbol{-k}}}}}}-{{{{{\boldsymbol{q}}}}}},x}-{u}_{{{{{{\boldsymbol{k}}}}}}y}{u}_{{{{{{\boldsymbol{-k}}}}}}-{{{{{\boldsymbol{q}}}}}},y}).$$

In the case of a finite nematic order parameter $\left\langle O\right\rangle \ne 0$, the degeneracy of the *E*_*g*_ phonons is lifted ${\omega }_{0}\to \sqrt{{\omega }_{0}^{2}\pm {g}_{{E}_{g}}\left\langle O\right\rangle }$, as the protecting symmetry is broken. The splitting of the squared frequencies is directly proportional to the order parameter.

While symmetry breaking is necessary to lift the degeneracy and to induce a nematic order parameter, it is not the only approach to achieve spectral splitting. Next, we show that this can indeed arise from a purely dynamical effect, which relates to the dynamic Jahn-Teller effect^34^, albeit rather originating from fluctuations of electronic degrees of freedom than from a large zero-point energy of vibronic modes. Similar phenomena have been reported in the half-field Holstein model^35^.

In order to get a qualitative understanding of such a dynamic splitting we consider a simple toy model that directly follows from our above description. We assume that *O*_*i*_ behaves as an effective Ising variable ${O}_{i}={A}_{0}{\tau }_{i}^{z}$, with typical amplitude *A*_0_. The Ising pseudospin states $\left|\Uparrow \right\rangle$ and $\left|\Downarrow \right\rangle$—defining the basis for the Pauli matrix *τ*^*z*^— describe the two orbital polarizations. Then the Hamiltonian takes the form

3 $${H}_{c,{E}_{g}}=\lambda \frac{{\omega }_{0}^{2}}{2}\mathop{\sum}\limits_{i}{\tau }_{i}^{z}({u}_{i,x}^{2}-{u}_{i,y}^{2})+\mathop{\sum}\limits_{i}\frac{{{\Omega }}}{2}{\tau }_{i}^{x}.$$

Here, $\lambda={g}_{{E}_{g}}{A}_{0}/{\omega }_{0}^{2}$ is a dimensionless nemato-elastic coupling constant. In the ordered state, the two modes become ${\omega }_{0}\to {\omega }_{0}\sqrt{1\pm \lambda \left\langle {\tau }^{z}\right\rangle }$ in agreement with the discussion above. Note that the model can be easily adapted to the coupling of *B*_2g_ nematicity, relevant for Fe-based superconductors, using *u*_*i*,*x*_. *u*_*i*,*y*_ instead of ${u}_{i,x}^{2}-{u}_{i,y}^{2}$ in the previous equation. This yields exactly the same results, albeit with a different strain dependence. From the splitting of the *E*_g_ phonons^8^ we can estimate *λ* ~ 0.08 for BaFe_2_As_2_, much weaker than *λ* ~ 0.7 of the Ni-system. To model quantum fluctuations, we introduced the last term that gives rise to tunneling processes between the two degenerate states^36^. Using a variational approach^37,38^, one can show that the tunneling rate can be thought of as a renormalized quantity ${{{\Omega }}}_{0}\to {{\Omega }}={{{\Omega }}}_{0}{e}^{-\int\nolimits_{0}^{{\omega }_{c}}\frac{{{{{{\rm{Im}}}}}}{{\Gamma }}(\omega )}{{(\omega+{{{\Omega }}}_{0})}^{2}}{{{\rm{d}}}}\omega }$ rooted in a more complex dynamic nematic susceptibility of the form ${\chi }_{{{{{{\rm{nem}}}}}}}({{\Omega }})={[{{{\Omega }}}_{0}^{2}-{\omega }^{2}+{{\Gamma }}(\omega )]}^{-1}$, similar to other pseudospin problems^39^.

**Fig. 4:**
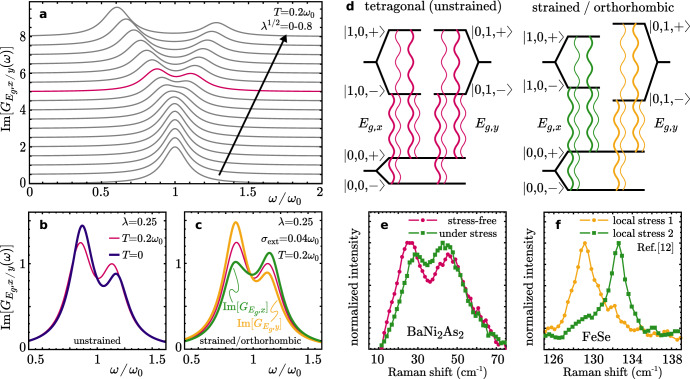
Model calculation. **a** Calculation of the *E*_g_Raman response using a simple model of two degenerate harmonic oscillators coupled via *λ* to a fluctuating nematic degree of freedom. Details of the calculation are laid out in the Supplementary Note 4. Parameters for the *B*_1g_ fluctuation frequency and temperature are chosen Ω = *ω*_0_/20, *T* = *ω*_0_/5. **b** The weight distribution of the peak splitting depends on the relative energy scales in the problem, as illustrated for two different temperatures *T* ≪ Ω and Ω ≪ *T* ≪ *ω*_0_. **c** In the disordered case with equal peak splitting the degeneracy of the two Raman responses can be lifted by applying a conjugate external strain *σ*_ext_. **d** Schematic of the allowed transitions that cause the peak splitting of the Raman signal even in the tetragonal state (left panel) and in the strained/orthorhombic state (right panel). **e** Raman response of BaNi_2_As_2_stress-free and under uniaxial stress and comparison to local stress dependence (**f**) of FeSe [Data from ref. 12, plotted with permission from the authors].

The electronic degrees of freedom will now give rise to some coupling of the ${\tau }_{i}^{z}$ at different lattice sites, responsible for actual nematic order. If we assume a mean field description of the tetragonal phase, different lattice sites decouple. Still ${H}_{{{{{{\rm{latt,}}}}}}{E}_{g}}+{H}_{c,{E}_{g}}$, that describes the *E*_g_ phonons, is a many-body model of interacting pseudospin and lattice vibrations. It is possible to obtain an exact solution of the model with many-body eigenenergies:

4 $${E}_{{m}_{1},{m}_{2},s}={\epsilon }_{+}({m}_{1}+{m}_{2}+1/2)+s\sqrt{{\epsilon }_{-}^{2}{\left({m}_{1}-{m}_{2}\right)}^{2}+\frac{{{{\Omega }}}^{2}}{4}},$$

where $2{\epsilon }_{\pm }={\omega }_{0}(\sqrt{1+\lambda }\pm \sqrt{1-\lambda })$, the indices *m*_1,2_ = 0, 1, 2, ⋯   refer to phonon occupations of the *E*_g,x_ and *E*_g,y_ modes, and *s* = ± 1 corresponds to the pseudospin states $\left|\pm \right\rangle=\frac{1}{\sqrt{2}}(\left|\Uparrow \right\rangle \pm \left|\Downarrow \right\rangle )$. From an analysis of the spectral functions follows that both phonon modes are degenerate and at T = 0 have two dominant peaks at *ω*_0±_ = *E*_1,0,±_ − *E*_0,0,±_ = *E*_0,1,±_ − *E*_0,0,±_ with splitting Δ*ω* = *ω*_+_ − *ω*_−_; see Fig. 4a, b. For *λ* = 0, *ω*_0+_ = *ω*_0−_ = *ω*_0_, i.e. there is no splitting. However, since the pseudospin splitting ${E}_{{m}_{1},{m}_{2},+}-{E}_{{m}_{1},{m}_{2},-}$ of the many-body eigenstates depends on the phonon-population via ${\epsilon }_{-}^{2}{({m}_{1}-{m}_{2})}^{2}$, both *E*_*g*_-modes split in two main satellites by ${{\Delta }}\omega=\sqrt{{{{\Omega }}}^{2}+2{\omega }_{0}^{2}(1-\sqrt{1-{\lambda }^{2}})}-{{\Omega }}$. For weak coupling *λ* ≪ Ω/*ω*_0_ (relevant for Fe-based systems) holds ${{\Delta }}\omega={\lambda }^{2}{\omega }_{0}^{2}/{{\Omega }}$ such that the splitting is small Δ*ω*/*ω*_0_ ≪ 1. However, as soon as *λ* is larger than Ω/*ω*_0_ we have Δ*ω* ≈ *λω*_0_ and the splitting of both E_*g*_-modes is of order unity (note that the system becomes unstable as *λ* → 1, which signals a structural instability).

To obtain a qualitative understanding of the origin of this behavior we use our earlier result for the static lifting $\sim \lambda \left\langle {\tau }^{z}\right\rangle {\omega }_{0}$ of the degeneracy and replace it by $\sqrt{{\lambda }^{2}\left\langle {({\tau }^{z})}^{2}\right\rangle }{\omega }_{0} \sim \lambda {\omega }_{0}$. This simply indicates that when the fluctuations between degenerate nematic configurations are strongly coupled and slow compared to the timescale of phonons (Ω ≲ *ω*_0_), a split spectral structure for the phonons similar to that induced by static ordering can be obtained. In Fig. 4a, b we also show the behavior at finite *T* where several additional satellites enter the analysis, but the overall behavior remains unchanged. Finally, we can include externally applied stress *σ*_ext_ that explicitly breaks the four-fold symmetry via ${H}_{\sigma }=-\!{\sum }_{i}{\sigma }_{{{{{{\rm{ext}}}}}}}{\tau }_{i}^{z}$. Now, the degeneracy of the two *E*_g_ phonons is lifted. We then observe merely a gradual transfer of weight between the split peaks, see Fig. 4c.

The precise nature of the orbital fluctuations and of the electronic state coupling to the phonon in BaNi_2_As_2_ remains to be clarified experimentally. Nevertheless, it is possible to experimentally investigate the symmetry-lifting for BaNi_2_As_2_, by performing measurements under strain, in a comparative study with FeSe. Quite generally, Fe-based superconductors are particularly soft and can be detwinned with very modest stress. This can be seen in a Raman experiment through the suppression in the intensity of one of the two degenerate *B*_2*g*_ or *B*_3*g*_ modes^9–12^. Here, we used the approach proposed in ref. 40, gluing a BaNi_2_As_2_ sample onto a glass-fiber reinforced plastic substrate with the edges of the tetragonal unit cell aligned with the fibers. The resulting symmetry breaking strain is estimated to 0.4% at 150 K, which decreases the triclinic transition temperature by about 5K and yields a small but measurable shift of the *A*_1g_ phonon of ~0.5 cm^−1^ (Supplemmentary Information). In sharp contrast to the Fe-based compounds (Fig. 4f), the intensity ratio between the two *E*_g_ features barely changes in BaNi_2_As_2_ (Fig. 4e), as we only a observe a small spectral weight transfer between the two satellites, in line with the above prediction, hereby confirming the dynamical nature of the nematic electronic phase of BaNi_2_As_2_.

## Discussion

**Fig. 5:**
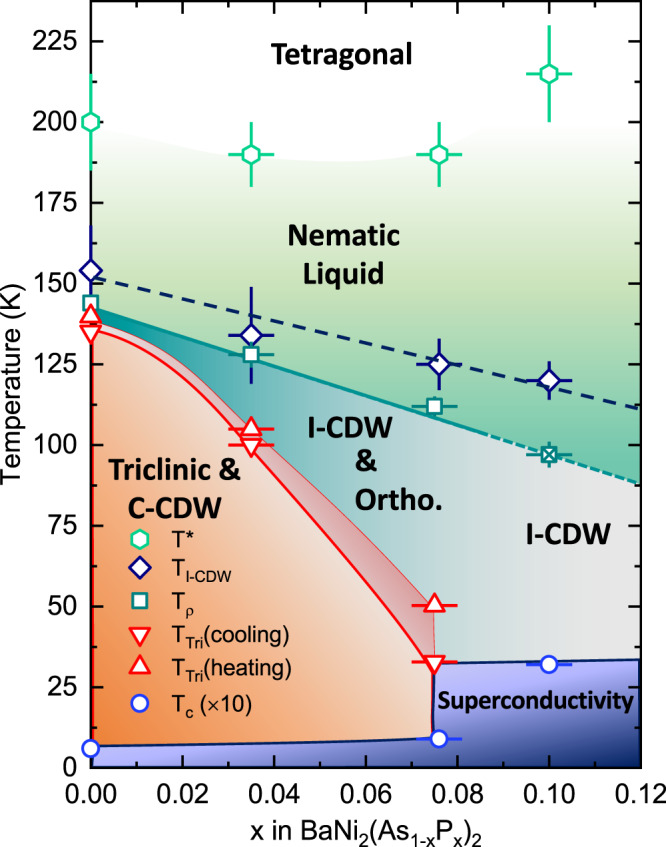
Phase diagram for ${{{{{\rm{Ba}}}}}}{{{{{{\rm{Ni}}}}}}}_{2}{({{{{{{\rm{As}}}}}}}_{1-x}{{{{{{\rm{P}}}}}}}_{x})}_{2}$. The transition temperatures for the triclinic phases are determined from transport and thermal expansion measurements. We also report the temperature *T*_*ρ*_ of the minimum in dR/dT which corresponds to an orthorhombic transition in the parent compound (Supplementary Note 2). The superconducting transition temperature is measured by specific heat (Supplementary Note 2). The onset of the C-CDW seen with XRD coincides with the triclinic transition, whereas the intensity of the I-CDW satellites increases strongly at *T*_I-CDW_, just above *T*_*ρ*_ (see Fig. 2). The onset of the broadening of the *E*_g,1_ Raman phonons is indicated by T^*^ (see also Supplementary Note 3). Horizontal error bars correspond to the uncertainty on the P-concentration as determined from EDX (Supplementary Note 1). Vertical error bars reflect the accuracy with which the various temperatures can be determined from Fig. 2.

We summarize our findings on the phase diagram shown in Fig. 5 in which we report the doping dependence of the characteristic temperatures of the BaNi_2_(As_1−*x*_)P_x_)_2_ system determined from a combination of XRD, Raman, resistivity and specific heat experiments. The main result of this study is the pronounced broadening and splitting of the *E*_g_ modes which occurs at temperatures significantly larger than that of static structural distortions and/or of the apparition of CDW orders. The effect is qualitatively and quantitatively very different from that associated with nematicity in Fe-based compounds. Indeed, rather than manifesting itself at a symmetry breaking phase transition or via long-wavelength fluctuations (probed e.g. through the softening of elastic constants or Raman scattering in the symmetry channel of the nematic order parameter), the behavior of the *E*_g_ modes can be explained by a strong symmetry-allowed coupling between the lattice and dynamic *B*_1g_ local nematic fluctuations visible even without phase transition. We argue that the *E*_g_ phonons follow the dynamics of an Ising variable, likely related to resonant transitions between distinct electronic orbital states, which causes a splitting in the phonon spectrum even in the absence of broken symmetry. These transitions can in principle be driven both by quantum and thermal fluctuations, and disentangling their effects is generally not trivial at finite temperature. We note however that the splitting of the *E*_g_ modes can be as large as ~30 cm^−1^. This effectively corresponds to a temperature scale of ~45 K, above which fluctuations between the two configurations of the system can in principle be thermally driven. This is most likely the case for most of the investigated samples, with the notable exception of the one containing 10% of phosphorus, in which triclinic transition is completely suppressed and *T*_c_ is enhanced. We note that the splitting amplitude remains essentially temperature independent below ~50 K (Supplementary Note 3), where quantum fluctuations can in principle be expected to take the lead, calling for further investigation on this interesting regime.

We end our discussion by drawing an analogy with generalized liquids states, strongly correlated states that do not break a symmetry, such as the Fermi^41^, orbital^42^ or the spin^43^ liquids. The regime of strong fluctuations between degenerate nematic states that splits the *E*_g_ phonons without breaking the rotational symmetry in BaNi_2_(As_1−*x*_)P_x_)_2_ discussed here can therefore be understood as a nematic liquid. The case of spin liquids in which strong zero-point fluctuations between degenerate configurations prevent long-range magnetic ordering^43^ might bear the strongest conceptual similarity with the present case. One can therefore expect that some of the phenomenology of the quantum spin liquid might carry over to nematic liquids and give rise to related phenomena, such as unconventional superconductivity or exotic quantum states with long-range entanglement. It strongly supports the view that the much-enhanced superconducting transition temperature of the studied materials upon doping is closely tied to the emergence of dynamic nematic fluctuations uncovered in our measurements.

## Methods

### Single crystal growth

Single crystals of ${{{{{\rm{Ba}}}}}}{{{{{{\rm{Ni}}}}}}}_{2}{({{{{{{\rm{As}}}}}}}_{1-x}{{{{{{\rm{P}}}}}}}_{x})}_{2}$ were grown using a self-flux method. NiAs binary was synthesized by mixing the pure elements Ni (powder, Alfa Aesar 99.999%) and As (lumps, Alfa Aesar 99.9999%) that were ground and sealed in a fused silica tube and annealed for 20 h at 730 °C. All sample handlings were performed in an argon glove box (O_2_ content <0.7 ppm). For the growth of ${{{{{\rm{Ba}}}}}}{{{{{{\rm{Ni}}}}}}}_{2}{({{{{{{\rm{As}}}}}}}_{1-x}{{{{{{\rm{P}}}}}}}_{x})}_{2}$, a ratio of Ba:NiAs:Ni:P = 1: 4(1 − *x*): 4*x*: 4*x* was placed in an alumina tube, which was sealed in an evacuated quartz ampule (*i.e*. 10^−5^ mbar). The mixtures were heated to 500 °C − 700 °C for 10 h, followed by heating slowly to a temperature of 1100 °C − 1150 °C, soaked for 5 h, and subsequently cooled to 995–950 °C at the rate of 0. 5 °C/h to 1 °C/h, depending on the phosphorus content used for the growth. At 950–995 °C, the furnace was canted to remove the excess flux, followed by furnace cooling. Plate-like single crystals with typical sizes 3 × 2 × 0.5 mm^3^ were easily removed from the remaining ingot. The crystals were brittle having shiny brass-yellow metallic lustre. Electron microscope analysis of the ${{{{{\rm{Ba}}}}}}{{{{{{\rm{Ni}}}}}}}_{2}{({{{{{{\rm{As}}}}}}}_{1-x}{{{{{{\rm{P}}}}}}}_{x})}_{2}$ crystals was performed using a benchtop scanning electron microscope (SEM) (Supplementary Note 1). The energy dispersive x-ray (EDX) analysis on the ${{{{{\rm{Ba}}}}}}{{{{{{\rm{Ni}}}}}}}_{2}{({{{{{{\rm{As}}}}}}}_{1-x}{{{{{{\rm{P}}}}}}}_{x})}_{2}$ crystals revealed phosphorus content *x* = 0.035 ± 0.005, 0.076 ± 0.005, and 0.10 ± 0.005.

### Single crystal X-ray diffraction

The phosphorus concentrations of the investigated samples were further confirmed by structural refinement from x-ray diffraction at room temperature using STOE imaging plate diffraction system (IPDS-2T) equipped with Mo K*α* radiation^20^. Detailed temperature dependencies of the I- and C-CDW superstructure peaks were obtained using a four circle diffractometer. The samples were cooled under vacuum in a DE-202SG/700K closed-cycle cryostat from ARS, surrounded by a Beryllium dome. The incoming beam was generated from a Molybdenum X-ray tube with a voltage of 50 kV and a current of 40 mA. The beam was collimated and cleaned up by a 0.8 mm pinhole before hitting the samples. We specifically followed the superstructure reflections close to the (4, 1, 1) and (1, 0, 3) Bragg peaks for the I- and C-CDW, respectively.

### Polarization-resolved confocal Raman scattering

Confocal Raman scattering experiments were performed with a Jobin-Yvon LabRAM HR Evolution spectrometer in backscattering geometry, with a laser power of ≤0.8 mW that was focused on the sample with a ×50 magnification long-working-distance (10.6 mm) objective. The laser spot size was ≈2 μm in diameter. Low-resolution mode (1.54 cm^−1^) of the spectrometer with 600 grooves/mm was used to maximize the signal output. For the phonon measurements, a He–Ne laser (*λ* = 632.8 nm) was used as the incident source, whereas the electronic background was best observed using the 532 nm line of a Nd:YAG solid state laser. Direct comparison of the structural phase transition temperature upon cooling as measured in specific heat and Raman indicated a laser-induced heating limited to less than 2K. The Raman spectra were Bose corrected and the phonons analyzed using a damped harmonic oscillator profile (with the exception of the *A*_1g_ mode that displayed a Fano asymmetry and was treated accordingly).

Additional details on the experiment, phonon calculations or on the theoretical model presented here are given in Supplementary Notes 3 and 4.

## Supplementary information

Supplementary Information

## Data Availability

The Raman data reported in this study have been deposited at the KIT Open, under the following identification number KITopen-ID: 1000148276. The data that support the findings of this study are available from the corresponding author, Matthieu Le Tacon, upon reasonable request.
