# Supplementary material for: An electronic nematic liquid in BaNi2As2
Source: Nat Commun. 2022 Aug 4;13:4535. doi: 10.1038/s41467-022-32112-7 (PMC9352674; doi:10.1038/s41467-022-32112-7)
Supplement: Supplementary file 1 — Supplementary Information [file 41467_2022_32112_MOESM1_ESM.pdf]

Supplementary Information: An Electronic Nematic Liquid in $BaNi_2As_2$

Yi Yao,[1] Roland Willa,[2] Tom Lacmann,[1] Sofia-Michaela Souliou,[1] Mehdi Frachet,[1] Kristin Willa,[1] Michael Merz,[1,3] Frank Weber,[1] Christoph Meingast,[1] Rolf Heid,[1] Amir-Abbas Haghighirad,[1] Jörg Schmalian,[1,2] and Matthieu Le Tacon[1]

[1]*Institut für Quantenmaterialien und -technologien,*

Karlsruher Institut für Technologie, 76021 Karlsruhe, Germany

[2]*Institut für Theorie der Kondensierten Materie,*

Karlsruher Institut für Technologie, 76131 Karlsruhe, Germany

[3]*Karlsruhe Nano Micro Facility (KNMFi), Karlsruhe Institute of Technology (KIT),*

76344 Eggenstein-Leopoldshafen, Germany

(Dated: July 12, 2022)

Supplementary Note 1. Single Crystal Composition Analysis

The composition of the investigated single crystals of $BaNi_2(As_{1-x}P_x)_2$ was determined by x-ray diffraction (as detailed in Ref. [1]) and energy dispersive x-ray (EDX) measurements. Those were performed using a Zeiss Auriga 60 scanning electron microscope equipped with an EDAX (Model Octane Elect) detector (x=0, x=0.035 ± 0.005 and x=0.076 ± 0.005) and a COXEM EM-30AXN SEM-EDX compact device equipped with an Oxford Instruments Aztec EDX System containing an EDX detector and AztecLiveLite Software (x = 0.10 ± 0.005).

Note that for the batch with P-concentration close to 7% small variations of the determined concentration were observed (within the accuracy of the EDX measurement which enables the determination of the P concentration within ±0.5%). Two samples and with $x = 0.073$ and $x = 0.076$ where investigated and displayed very similar behavior. The corresponding resistivity measurements were carried out on two samples from the same batch for which the determined concentration were 0.070 and 0.075, respectively. A clear reduction of the triclinic transition temperature was observed but no difference in the phononic behavior was noticed. Typical electron micrograph images are shown for the x = 0.10 sample in Supplementary Figure 1, which also shows the element distribution maps. The corresponding EDX spectrum is also shown.

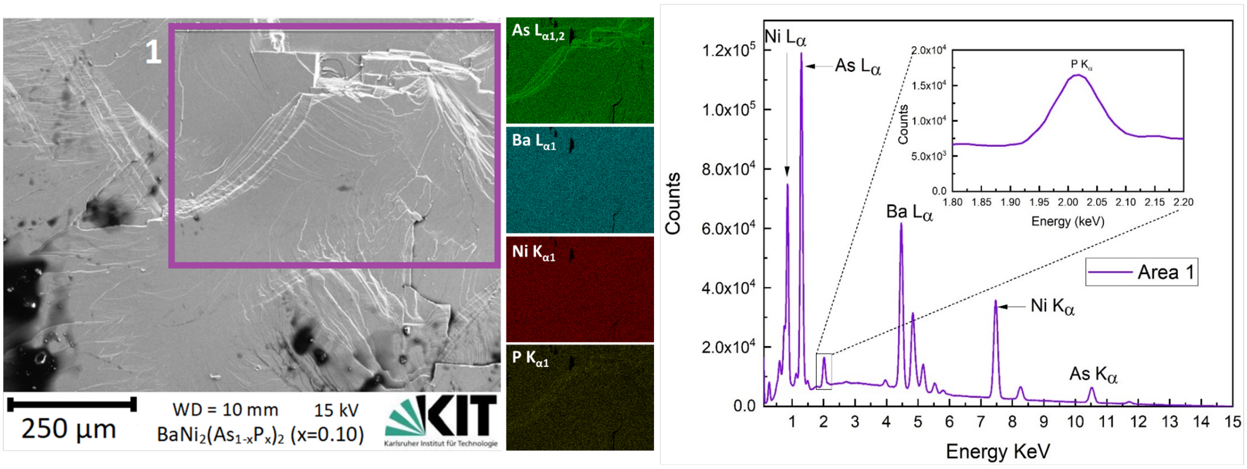

Supplementary Fig. 1: EDX. (left) Electron micrograph image of the x=0.10 $BaNi_2(As_{1-x}P_x)_2$sample, with the EDX maps for the different elements and (right) the integrated spectrum for the representative area 1 in the image.

Supplementary Note 2. Transport and thermodynamic measurements

1. Dilatometry and transport

Electrical resistance was measured in standard 4-wires geometry, either using a combination of LakeShore 6221 current source and LakeShore 2182A Nanovoltmeter, or using a high precision resistance bridge (LakeShore 372). Typical samples dimensions were 2mm x 1mm x 50 μm. Resistance was measured along the a crystallographic direction of the tetragonal unit cell. A slow cooling rate of about 1K to 1.5K was used to cool down the single crystals. To avoid any unwanted external stress on the sample, measurements were performed under fully free-standing conditions.

In Supplementary Figure 2-a, we compare the resistivity of the $BaNi_2(As_{1-x}P_x)_2$ sample with x=0.035 to high-resolution thermal expansion $\Delta L/L$ obtained on a similar sample (upon warming). Details for the dilatometry measurements are given in ref.[1]. The dilatometry measured along the [100] and the [110] direction (in the high-T tetragonal notation) shows a pronounced first-order transition below T_{Tri} = 105 K. Below T_ρ = 127 K, a clear difference between the $\Delta L/L$ measured along the two directions is seen and disappear gradually. This evidences a second order transition from a weakly distorted orthorhombic phase that exists above T_{Tri} and up to T_ρ, above which the tetragonal phase is restored.

In Supplementary Figure 2-b, we show that the splitting of this orthorhombic onset coincides with the broad minimum in the temperature derivative of the resistivity dR/dT. Note that this coincidence seems to hold primarily at low doping. Preliminary dilatometry investigation in the 7.6% sample indicate that the orthorhombic transition occurs at a temperature lower than T_ρ and could not be detected in the 10% sample, even though the system displays an I-CDW and a minimum in dR/dT consistent with the other samples (hence the different symbol for T_ρ for this sample in Fig. 5). This will be investigated further independently but does not affect any of the conclusions of the present work. A complete thermodynamic study of $BaNi_2(As_{1-x}P_x)_2$is presented in ref.[2].

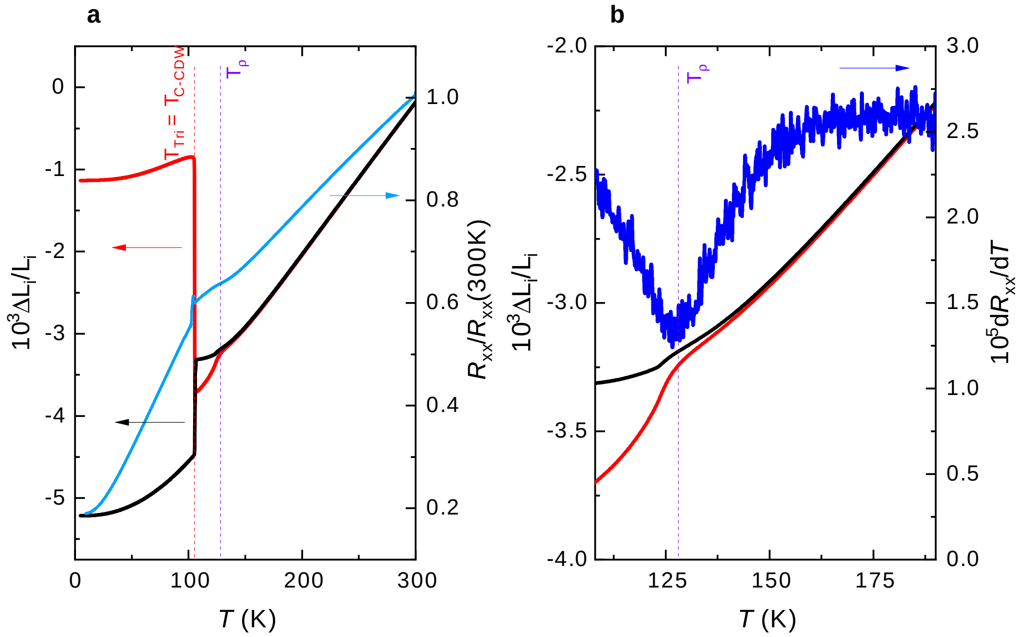

Supplementary Fig. 2: Resistance and thermal expansion of $BaNi_2(As_{1-x}P_x)_2$ single crystals with $x = 0.035$. (a) Temperature dependence of room-temperature normalized resistance (light blue) and thermal expansion (red for [100] crystallographic direction, black for [110] direction) both measured upon warming. At a given substitution the analogous triclinic transition temperatures establish that similar samples have been investigated by both experimental techniques. (b) Limited temperature range around the orthorhombic distortion temperature. The resistance is replaced by its temperature derivative (blue). The broad minimum in dR/dT coincides well with the orthorhombic splitting onset resolved in thermal expansion.

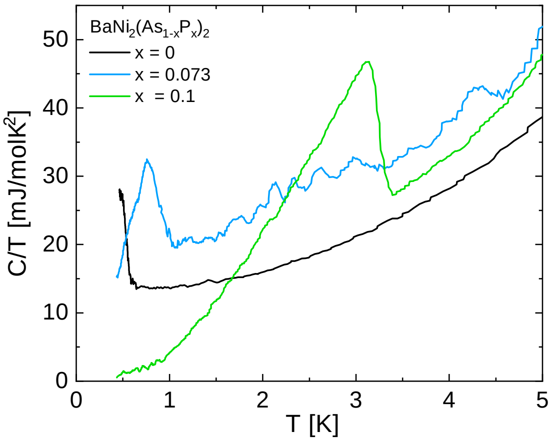

Supplementary Fig. 3: Heat capacity measurement showing a jump at the superconducting transition temperature T_c for $BaNi_2(As_{1-x}P_x)_2$ with $x = 0$, 0.073, and 0.1.

2. *Heat Capacity*

To measure the superconducting T_c, we have mounted the $BaNi_2(As_{1-x}P_x)_2$samples ($x = 0, 0.073$ and 0.1) on a He-3 probe allowing for measurements down to 0.4K. The heat capacity was then measured with the Physical Properties Measurement System (PPMS) from Quantum Design using the dual slope method. The sample was attached to the specific heat platform with Apiezon N grease. An addenda measurement was taken beforehand and subtracted from the sample measurement later. Typical measurements are shown in Supplementary Figure 3.

Supplementary Note 3. Raman scattering

Raman scattering measurements were performed on single crystals of $BaNi_2(As,P)_2$ with the low-resolution mode of a single grating Jobin-Yvon Labram spectrometer (600 grooves/mm grating) to maximize the signal output. Phonon measurements were done with a He-Ne laser (633 nm). The Raman-active optical modes of $BaNi_2(As,P)_2$ were determined following the *nuclear site group analysis*. Each unit cell contains one Ba-, two As- and two Ni-atoms, resulting in 15 zone-center vibrational modes. In Supplementary Table I we summarize the site symmetry of each point, the resulting normal modes, and the Raman-active optical modes within each phase.

Tetragonal phase ($I4/mmm$)		
Atom	Site symmetry	Irreducible representations
Ba	D_{4h}	$1A_{2u} + 1E_u$
As	C_{4v}	$1A_{1g} + 1A_{2u} + 1E_u + 1E_g$
Ni	D_{2d}	$1A_{2u} + B_{1g} + 1E_u + 1E_g$
Γ_{Raman}		$1A_{1g} + 1B_{1g} + 2E_g$
Orthorhombic phase (I/mmm)		
Atom	Site symmetry	Irreducible representations
Ba	D_{2h}	$1B_{1u} + 1B_{2u} + 1B_{3u}$
As	C_{2v}	$1A_g + 1B_{1u} + 1B_{2g} + 1B_{2u} + 1B_{3g} + 1B_{3u}$
Ni	C_{2v}	$1A_g + 1B_{1u} + 1B_{2g} + 1B_{2u} + 1B_{3g} + 1B_{3u}$
Γ_{Raman}		$2A_g + 2B_{2g} + 2B_{3g}$
Triclinic phase (P-1)		
Atom	Site symmetry	Irreducible representations
Ba	C_1	$3A_u$
As	C_1	$3A_g + 3A_u$
Ni	C_1	$3A_g + 3A_u$
Γ_{Raman}		$6A_g$

Supplementary Tab. I: Atomic site symmetries and the resulting normal modes for each point and the Raman-active optical modes Γ_{Raman} for $BaNi_2(As,P)_2$ in different phases.

1. *Group theory and polarization selection rules*

At room temperature, $BaNi_2(As,P)_2$ possesses the tetragonal structure with space group $I4/mmm$ and point group D_{4h}, thus $1A_{1g}$, $1B_{1g}$ and $2E_g$ modes are expected.

Upon cooling, the system undergoes a second-order orthorhombic transition and the space group changes to I/mmm with point group D_{2h}[1]. By breaking the four-fold symmetry, the A_{1g} and B_{1g} modes of the tetragonal phase become A_g modes in the orthorhombic phase, and the doubly degenerate E_g modes of the tetragonal phase split into nondegenerate B_{2g} and B_{3g} modes in the orthorhombic phase. However, the amplitude of the orthorhombic distortion is

Irreducible representations	A_{1g}	B_{1g}	E_g
Raman tensor	$\begin{pmatrix} a & 0 & 0 \\ 0 & a & 0 \\ 0 & 0 & b \end{pmatrix}$	$\begin{pmatrix} c & 0 & 0 \\ 0 & c & 0 \\ 0 & 0 & 0 \end{pmatrix}$	$\begin{pmatrix} 0 & 0 & 0 \\ 0 & 0 & e \\ 0 & e & 0 \end{pmatrix}$
Polarization selection rule	XX, YY, ZZ	XX, YY	XZ, YZ

Supplementary Tab. II: Raman tensor and polarization configuration of Raman-active modes of $BaNi_2(As_{1-x}P_x)_2$

very small (in the order of 10^{-4})[1], as shown by the thermal expansion and XRD measurements, therefore, no drastic modifications in the phonon position or linewidth are expected across T_ρ. Finally below the first-order transition temperature T_{Tri}, the symmetry of the structure is further lowered to triclinic with space group $P\bar{1}$ and point group C_i, where all phonon modes have A_g symmetry. The Raman tensor and polarization configuration for each Raman-active mode of the tetragonal phase is shown in Supplementary Table II.

Theoretical estimations of the phonon energies are given in Supplementary Note 3.2. Note that due to the small amplitude of the orthorhombic distortion and the narrow temperature window of the orthorhombic phase, the phonon frequencies are expected to be close to those in the tetragonal phase.

2. *Phonon energy calculation*

Lattice dynamics properties for the for the different structures of $BaNi_2As_2$ structure were calculated using the linear response or density-functional perturbation theory (DFPT) implemented in the mixed-basis pseudopotential method.

The electron-ion interaction is described by norm-conserving pseudopotentials, which were constructed following the descriptions of Hamann, Schlüter, Chiang[3,4] for Ba and Vanderbilt[5] for Ni and As, respectively. Semi-core states Ba-5p, Ni-3s, Ni-3p were included in the valence space. In the mixed-basis approach, valence states are expanded in a combination of plane waves and local functions at atomic sites, which allows an efficient description of more localized components of the valence states. Here, plane waves with a cut-off for the kinetic energy of 22 Ry and local functions of p,d type for Ba and s, p, d type for Ni, respectively, were

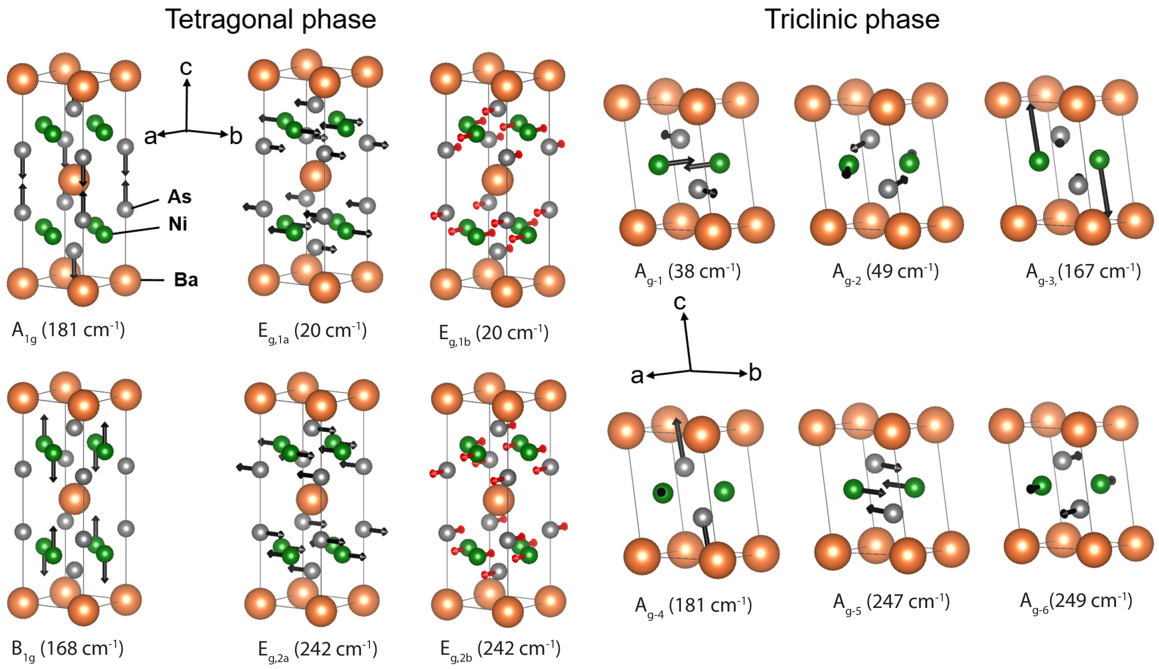

Supplementary Fig. 4: Schematic representations of the Raman-active modes of $BaNi_2As_2$ both in the high-temperature tetragonal and the low-temperature triclinic phases. Phonon eigenvectors are represented by black arrows (the red arrows show degenerate modes), and corresponding irreducible representations and phonon frequencies (with internal structural parameters relaxed) are written below each figure.

employed. Brillouin-zone integration was performed by sampling a tetragonal $16 \times 16 \times 8$ k-point mesh in conjunction with a Gaussian broadening of 100 meV. The exchange-correlation functional was represented by the general-gradient approximation in the PBE form[6]. Structural parameters were taken from room-temperature XRD measurements of the tetragonal phase ($a = 4.1413$ Å, $c = 11.6417$ Å) and from measurements taken at 90 K in the triclinic phase ($a = 4.1405$ Å, $b = 4.1538$ Å, $c = 6.4587$ Å, $\alpha = 108.678°$, $\beta = 108.657°$, $\gamma = 90.023°$). Internal stuctural parameters were relaxed until the atomic forces were smaller than 2.6×10^{-2} eV/Å. Eigendisplacements and energies of the Raman active phonons for the tetragonal and triclinic phases are summarized in Supplementary Figure 4.

Additional phonon calculations were performed for the orthorhombic $Immm$ structure of $BaNi_2As_2$ based on experimental data taken at 140 K ($a = 4.1254$ Å, $b = 4.129$ Å, $c = 11.646$ Å). For both E_g modes, the symmetry reduction induced by the orthorhombic distortion leads to

splittings of less than 0.3 cm^{-1}.

3. *Fitting details*

The Raman spectra in the XZ geometry were fitted between 10 cm^{-1} and 1220 cm^{-1}. To subtract the continuum a background with the analytical formula

$$\chi''_{\mathrm{bgd}} = (\alpha_1 + \alpha_2 T)\tanh\left(\frac{\Omega}{\tilde{\Gamma}_0(T)}\right) + (\beta_1 + \beta_2 T)\left(\frac{\Omega}{\tilde{\Gamma}_0(T)}\right) \tag{1}$$

which obeys causality was used[9]. The parameters $\alpha_1, \alpha_2, \beta_1, \beta_2$ are fixed for one doping value and were extracted from an earlier fitting step with free $\alpha_1(T)$ and $\beta_1(T)$. Unlike in the Raman study of $Ba(Fe_{1-x}Co_x)_2As_2$ from ref. 9 in our case $\tilde{\Gamma}_0(T)$ cannot easily be correlated to $\rho(T)$ such that $\tilde{\Gamma}_0(T)$ is chosen as free parameter. The full fitting function contained up to 8 damped harmonic oscillator profile to describe $E_{g,1a}, E_{g,1b}, E_{g,2a}, E_{g,2b}$, three additional features between 90 cm^{-1} and 200 cm^{-1} growing in intensity close to $T_{\mathrm{I-CDW}}$ and an feature around 325 cm^{-1} observed in the higher doped samples. Additionally, a resolution limited artifact from the notch filter around 15 cm^{-1} was removed by adding a Gaussian line shape to the fit function. The background and damped harmonic oscillator profiles were weighted by $\left(1-\exp(-\frac{\mathrm{hc}\omega}{\mathrm{k_B}T})\right)^{-1}$ to

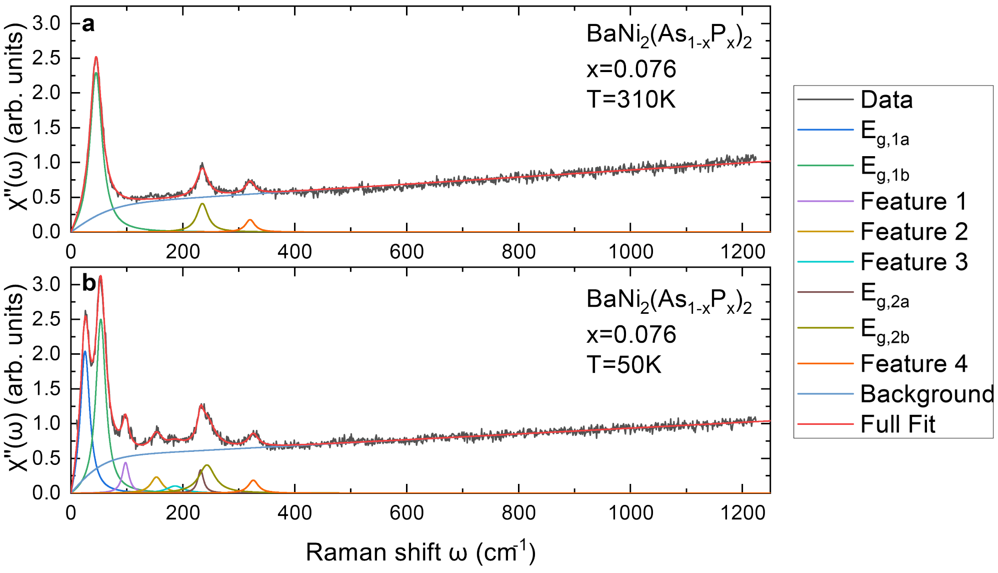

Supplementary Fig. 5: Raman spectrum with subtracted filter line and Bose factor correction with the full fit and individual fit components (a) above the splitting (310 K) and (b) below the splitting temperature (50 K) of $BaNi_2(As_{1-x}P_x)_2$ with $x = 0.076$.

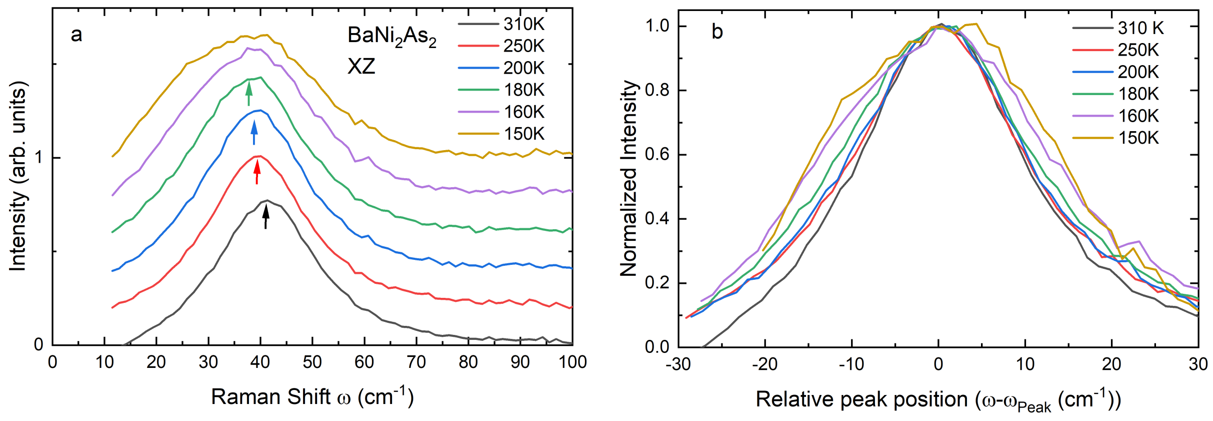

Supplementary Fig. 6: a) low energy part of the XZ Raman spectra of $BaNi_2As_2$ for selected temperatures. b) Broadening of the $E_{g,1}$ phonon of $BaNi_2As_2$ upon cooling.

account for the Bose factor correction.

In Supplementary Figure 5 exemplary of the fits above the splitting (310 K) and well below the splitting (50 K) of the 7.6% doped sample are shown. Above the splitting temperature only one damped harmonic oscillator profile for each E_g is used. Below the splitting temperature a second damped harmonic oscillator profile is added to distinguish $E_{g,1a}$ and $E_{g,1b}$ while for $E_{g,2}$ only for $x = 0.076$ and $x = 0.10$ a second damped harmonic oscillator profile was added.

To check the robustness of the fit different approaches for the background were tested all giving comparable peak positions/splitting and relative intensities.

4. E_g mode broadening, splitting and determination of T^*

The most spectacular result of our study is the large splitting of the E_g phonons upon cooling. To determine the onset temperature of the effect, T^* in the phase diagram shown in Fig. 5 of the main text, we have employed two methods which yield very similar results. On the one hand, T^* corresponds to the onset of the broadening to the $E_{g,1}$ mode upon cooling, which can be directly extracted from the fitting of the data. In Supplementary Figure 6-a, we show the (background subtracted) low energy data taken in $BaNi_2As_2$ with the XZ polarization, which already evidences the anomalous softening of the mode with decreasing temperature. The $E_{g,1}$ phonons have been aligned by shifting the spectra relative to the energy of the mode and their intensity normalized, from which a broadening is clearly visible already at 180 K (Fig. S6-b). The linewidth (full width at half maximimum) of the $E_{g,1}$ is ploted as function of temperature

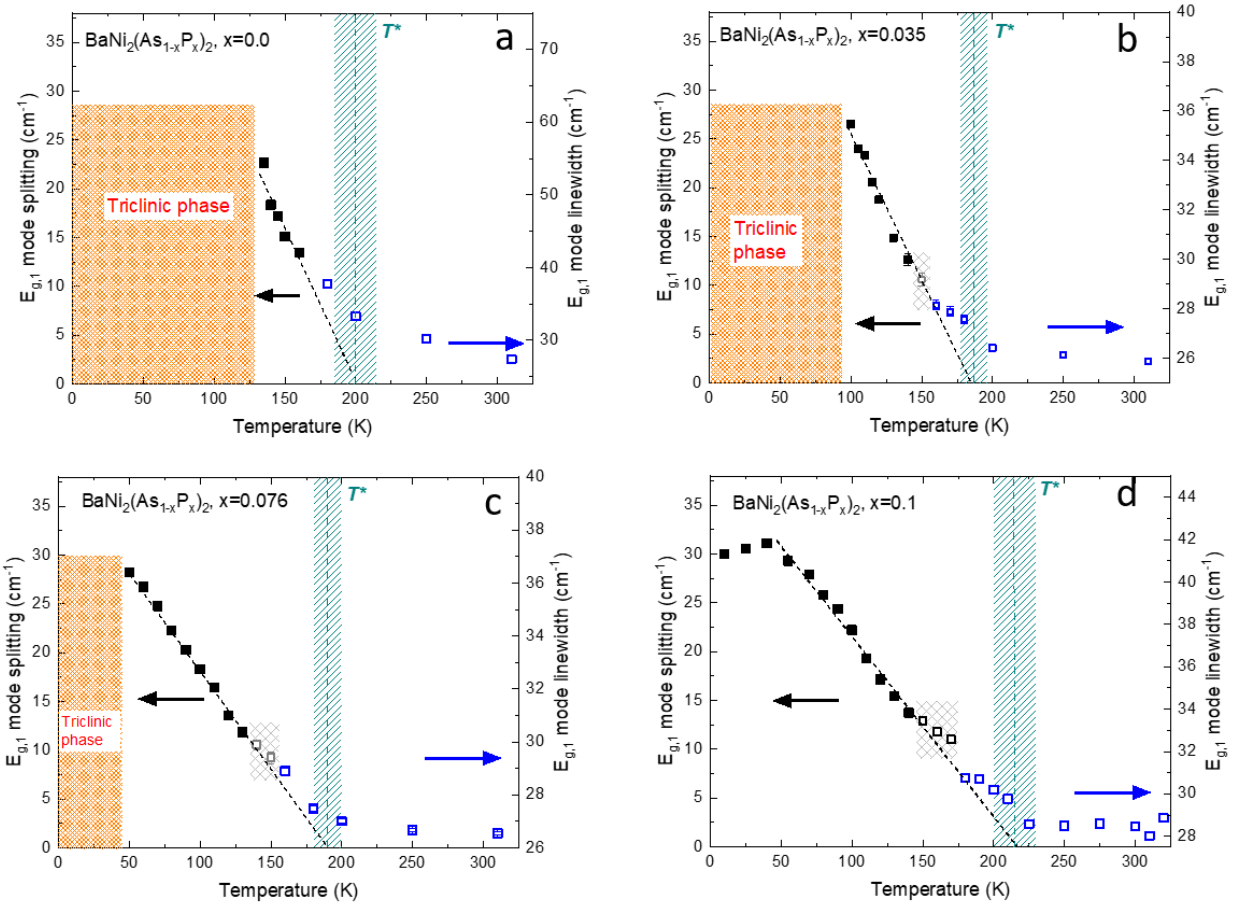

Supplementary Fig. 7: a) Temperature dependence of the splitting amplitude (black points) and broadening (blue points) of the $E_{g,1}$ mode of $BaNi_2As_2$. The former are obtained from the two mode fitting (see e.g. Supplementary Figure 5-b), whereas the latter data is obtained form a single line fit (Supplementary Figure 5-a). b) same for $BaNi_2(As_{1-x}P_x)_2$(x=0.035) c) same for $BaNi_2(As_{1-x}P_x)_2$(x=0.076) d) same for $BaNi_2(As_{1-x}P_x)_2$(x=0.1). In the panels b, c and d, the grey shaded area corresponds to the temperature region where the two lines cannot be fully resolved.

in Supplementary Figure 7 for the four investigated samples. At temperatures low enough, the splitting is unambiguous (the grey shaded areas of panels b,c and d of that figure correspond to regions where the phonon lineshape deviates from standard single damped harmonic oscillator and can already be fitted with two modes, even though the two peaks cannot be resolved) and increases linearly as temperature decreases. When extrapolating this linear dependence to high temperatures, we find that the temperature at which the splitting vanishes corresponds well to T^* determined from the broadening.

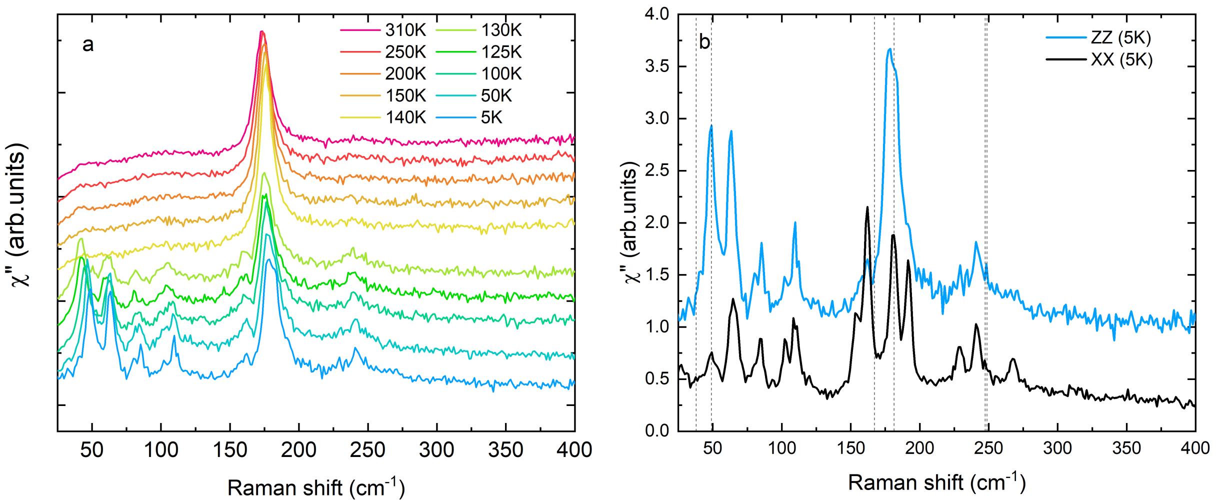

Supplementary Fig. 8: a) Temperature dependence of the ZZ Raman spectra of $BaNi_2As_2$. All spectra are scaled and shifted vertically for clarity. b) XX and ZZ Raman spectra of $BaNi_2As_2$ at 5K. The dashed lines represent the calculated phonon frequencies for the triclinic phase (see Supplementary Note 3.2).

5. *Spectra in the triclinic phase*

The symmetry reduction of across the first order structural transition is accompanied with a strong renormalization of the vibrational spectra. This is well illustrated by the spectra obtained in the ZZ polarization configuration, which probes the A_g symmetry.

As discussed in Supplementary Note 3.1, only 6 Raman-active modes with the A_g symmetry are expected below the first-order triclinic transition. However, as can be seen in Supplementary Figure 8, much more modes can be resolved in the triclinic phase and are related to the formation of the commensurate CDW superstructure. Note that we also observe a significant renormalization of the electronic background across the triclinic transition over a broad spectral range, which will be discussed in a future publication.

6. *Raman measurements under stress*

The behavior of the E_g modes in strained pure $BaNi_2As_2$ is discussed in the main text. A crystal of $BaNi_2As_2$ was broken in half, and we glued one of half on a glass-fiber-reinforced plastic (GFRP) substrate that was used to detwin $BaFe_2As_2$ in thermal expansion measurements[8]. The glue was chosen to be the Devcon 5 Minute Epoxy to ensure strong adhesion. The other

half was glued on a copper piece with high-vacuum Apiezon N Grease as strain-free reference. The mounting configuration is shown in Supplementary Figure 9. The sample was required to be very flat and thin for a homogeneous strain field along the crystallographic *c*-axis. The width and height of the fiberglass were specifically cut to 1 mm, and the whole piece of fiberglass was glued on the cold finger of our cryostat with Apiezon to ensure good thermal contact. The solid lines in Supplementary Figure 9(a) indicate the fiber direction. The thermal expansion coefficient along this direction is larger than that perpendicular to it as shown in the inset of Supplementary Figure 9, thus under cooling, the sample is subjected to a symmetry-breaking strain $|\epsilon_a - \epsilon_b| = |\Delta L_{\|}/L_{\|}^{300K} - \Delta L_{\perp}/L_{\perp}^{300K}|$ with a magnitude of $\sim 4.10^{-3}$ at 150 K.

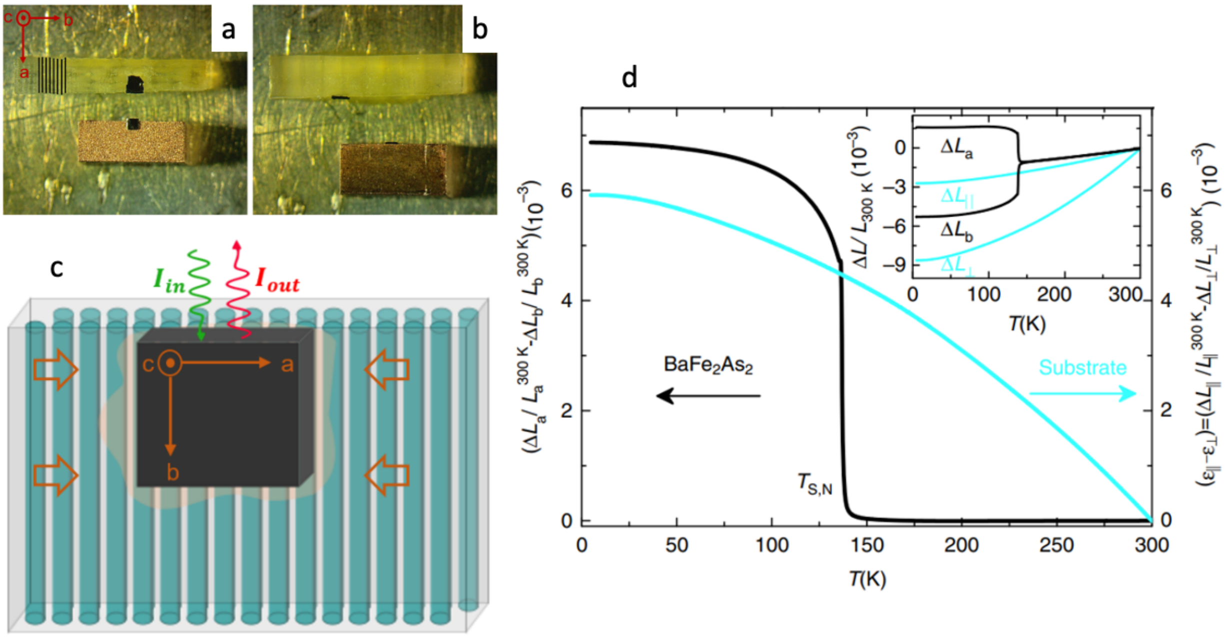

Supplementary Fig. 9: Schematic diagram of the crystal mounting on the fiberglass and copper stage. (a) In-plane demonstration. The vertical lines indicate the direction of the fibers. (b) Edge measurement configuration for the XZ/YZ polarization. (c) Scattering geometry for the Raman measurements. (d) The anisotropic strain of the GFRP substrate in comparison to the in-plane orthorhombic distortion of a free-standing $BaFe_2As_2$. The thermal expansion is plotted in the inset. The figure is taken from Ref. [8].

A small but measurable shift of the phonon lines of the strained sample compared to the unstrained one can be seen. The amplitude of the shift naturally depends on the mode considered. In the case of the A_{1g} phonon seen in ZZ or XX (see Supplementary Figure 10-a), it typically amounts to 0.5 cm^{-1}. Monitoring the renormalization of the phonons across the

first order transition (or the apparition of the additional peaks of the low temperature phase) allows us to estimate that T_{Tri} is reduced by 5K under strain (Supplementary Figure 10-b).

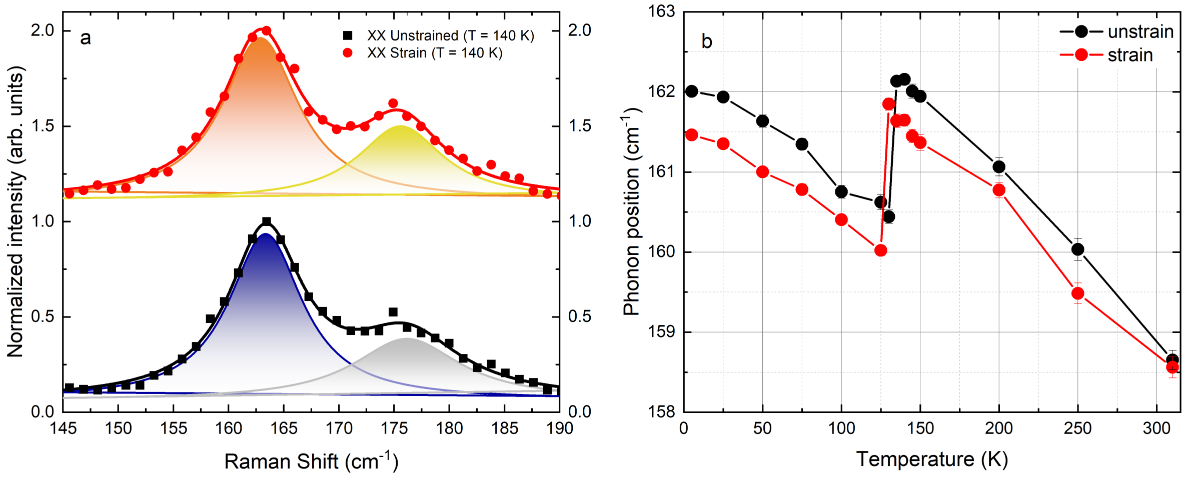

Supplementary Fig. 10: Raman measurement with and without strain (a) Comparison of the XX Raman response of $BaNi_2As_2$ at T = 140 K with and without strain (b) temperature dependence of the A_{1g} phonon energy of $BaNi_2As_2$ with and without strain.

Supplementary Note 4. Theoretical Model

1. *Raman response and Lehmann representation*

We start from the simple model layed out in the main text, where two degenerate harmonic modes (phonons) with E_g symmetry couple to an Ising variable $|s\rangle \in \{|\Uparrow\rangle = (1,0), |\Downarrow\rangle = (0,1)\}$ capturing the nematic degree of freedom. In addition, the systems' degeneracy can be externally lifted by coupling to external stress $\sigma_{\rm ext}$ that plays the role of a a conjugate field to the Ising variable, via $-\sigma_{\rm ext}\tau_z$ Here and below, the Pauli matrices τ_α act in the space of the pseudospin $|s\rangle$. Using the approach of Ref. 7, the Raman response in the B_{1g} is given by $R_{B_{1g}}(\omega) = -[n(\omega)+1]\text{Im}[D_{B_{1g}}(\omega)]/\pi$, where $n(\omega)$ is the Bose distribution function and $D_{B_{1g}}(\omega) \equiv \langle\langle \tau_z;\tau_z\rangle\rangle_\omega$ is the Fourier transform of the retarded function $D_{B_{1g}}(t-t') = -i\theta(t-t')\langle[\tau_z(t),\tau_z(t')]\rangle$. For the E_g modes we want to determine

$$D_{E_g,x}(\omega) = \langle\langle u_x;u_x\rangle\rangle_\omega \tag{2}$$

$$D_{E_g,y}(\omega) = \langle\langle u_y;u_y\rangle\rangle_\omega \tag{3}$$

which are the Fourier transform of

$$D_{E_g,\alpha}(t-t') = -i\theta(t-t')\langle[u_\alpha(t), u_\alpha(t')]\rangle. \tag{4}$$

with $\alpha = x, y$. Without nematic order the two E_g Green's functions are identical.

In the coupled problem it is better to use the Lehmann representation of the Green's function and determine the eigenvalues and matrix elements. To this end we consider a generic Green's function $G^r_{A,B}(t,t') = \langle\langle A(t); B(t')\rangle\rangle^r \equiv -i\theta(t-t')\langle[A(t), B(t')]\rangle$, and introduce the Fourier transform of the correlation functions

$$\langle B(0)A(t)\rangle = \int_{-\infty}^{\infty} \frac{d\omega}{2\pi} J(\omega) e^{-i\omega t}. \tag{5}$$

After a bit of algebra follows

$$J(\omega) = \frac{2\pi}{Z} \sum_{\ell,m} e^{-\beta E_m} \langle\ell|A|m\rangle\langle m|B|\ell\rangle\delta(\omega + E_\ell - E_m), \tag{6}$$

where $\{|\ell\rangle\}$ are the exact eigenfunctions of the Hamiltonian with eigenvalues $\{E_\ell\}$, i.e. $H|\ell\rangle = E_\ell|\ell\rangle$ and $Z = \sum_\ell e^{-\beta E_\ell}$ is the partition sum. In our case holds $A = B$ and get access to the propagator after determining the spectral function since $J(\omega) = -2n(\omega)\mathrm{Im}G^r_{AA}(\omega)$.

2. *Diagonalization of the Hamiltonian*

In order to analyze the coupled problem assume without restriction that $M = 1$, and introduce $\nu_\pm = \sqrt{\omega_0^2 \pm \lambda^2}$, $f_\pm = \frac{1}{2}(\sqrt{\nu_+} \pm \sqrt{\nu_-})$, and $g_\pm = \frac{1}{2}(\frac{1}{\sqrt{\nu_+}} \pm \frac{1}{\sqrt{\nu_-}})$. This allows us to express the momenta p_j $(j = x, y)$ and the displacements u_j in terms of bosonic latter operators

$$p_x = \frac{i}{\sqrt{2}}(f_+ + f_-\tau_z)(a_x^\dagger - a_x) \qquad \nu_x = \frac{1}{\sqrt{2}}(g_+ + g_-\tau_z)(a_x^\dagger + a_x) \tag{7}$$

$$p_y = \frac{i}{\sqrt{2}}(f_+ - f_-\tau_z)(a_y^\dagger - a_y) \qquad \nu_y = \frac{1}{\sqrt{2}}(g_+ - g_-\tau_z)(a_y^\dagger + a_y) \tag{8}$$

Using $f_+g_+ + f_-g_- = 1$ and $f_+g_- + f_-g_+ = 0$ it follows that $[\nu_j, p_k] = i\delta_{jk}$ is indeed obeyed if $[a_j, a_k^\dagger] = \delta_{jk}$ are ordinary bosonic operators. The original Hamiltonian now reads

$$H = (\mu_+ + \mu_-\tau_z)(a_x^\dagger a_x + \frac{1}{2}) + (\mu_+ - \mu_-\tau_z)(a_y^\dagger a_y + \frac{1}{2}) + \frac{\Omega}{2}\tau_x - \sigma_{\mathrm{ext}}\tau_z \tag{9}$$

with and $\mu_\pm = (\nu_+ \pm \nu_-)/2$. The last two terms remain unaffected by the transformation. In the basis $|\ell_x, \ell_y\rangle$ of the harmonic oscillator, the Hamiltonian assumes a block-diagonal form with

$$\langle\ell_x\ell_y|H|m_x m_y\rangle = \delta_{\ell_x m_x}\delta_{\ell_y m_y}[\epsilon_{\ell_x\ell_y}\tau_0 + h_{\ell_x\ell_y}\tau_z + (\Omega/2)\tau_x] = \delta_{\ell_x m_x}\delta_{\ell_y m_y}H_{\ell_x\ell_y}. \tag{10}$$

The parameters $\epsilon_{\ell_x\ell_y} = \mu_+(\ell_x + \ell_y + 1)$ and $h_{\ell_x\ell_y} = \mu_-(\ell_x - \ell_y) - \sigma_{\text{ext}}$ depend on the quantum numbers of the harmonic fields. In order to diagonalize the remaining two-level system it is important to keep track on the sign of $h_{\ell_x\ell_y}$. The unitary transformation

$$U_{\ell_x\ell_y} = U(\vartheta_{\ell_x,\ell_y}) \equiv \begin{pmatrix} \cos(\vartheta_{\ell_x,\ell_y}/2) & -\sin(\vartheta_{\ell_x,\ell_y}/2) \\ \sin(\vartheta_{\ell_x,\ell_y}/2) & \cos(\vartheta_{\ell_x,\ell_y}/2) \end{pmatrix} \tag{11}$$

consistently transforms the Hamiltonian into a diagonal form $\tilde{H}_{\ell_x\ell_y} = U^\dagger_{\ell_x\ell_y} H_{\ell_x\ell_y} U_{\ell_x\ell_y} = \text{diag}[\Lambda^+_{\ell_x\ell_y}, \Lambda^-_{\ell_x\ell_y}]$ with $\Lambda^\pm_{\ell_x\ell_y} = \mu_+(\ell_x+\ell_y+1) \pm \{[\mu_-(\ell_x-\ell_y)-\sigma_{\text{ext}}]^2+(\Omega/2)^2\}^{1/2}$ $[\Lambda^+_{\ell_x\ell_y} > \Lambda^-_{\ell_x\ell_y}]$, and $\vartheta_{\ell_x,\ell_y} = \arctan(h_{\ell_x\ell_y}, \Omega/2)$. This generalized form of the arctan function takes into account in which quadrant the point $(h_{\ell_x\ell_y}, \Omega/2)$ lies [it holds that $\arctan(0, \Omega/2) = \pi/2$].

In the fully diagonalized basis $\{|\ell_x\ell_y\sigma\rangle \mid \ell_j \in \mathbb{N}_0,\ \sigma = \pm\}$, the energy reads

$$\begin{aligned} \langle \ell_x\ell_y\sigma|H|m_x m_y\sigma'\rangle &= \delta_{\ell_x m_x}\delta_{\ell_y m_y}\delta_{ss'}\left\{\mu_+(\ell_x+\ell_y+1) + \sigma\sqrt{[\mu_-(\ell_x-\ell_y)-\sigma_{\text{ext}}]^2 + (\Omega/2)^2}\right\} \\ &= \delta_{\ell_x m_x}\delta_{\ell_y m_y}\delta_{ss'}E_{\ell_x,\ell_y,\sigma}. \end{aligned} \tag{12}$$

Any other observable can be transformed into the Hamiltonian's eigenbasis. An observable A transforms as $\tilde{A} = U^\dagger_{\ell_x\ell_y} A U_{\ell_x\ell_y}$ where the unitary transformation in the pseudospin $|\sigma\rangle \in \{|\pm\rangle\}$ basis depends on the occupation operators ℓ_i of the harmonic oscillators. We are now ready to

compute $J(\omega)$. Following Eq. (6) we find

$$J_{E_g,x}(\omega) = \tag{13}$$

$$\frac{\pi}{Z}\sum_{\ell_x,\ell_y}\sum_{\sigma} e^{-\beta E_{\ell_x,\ell_y,\sigma}}\Big\{ (\ell_x+1)\delta(\omega+E_{\ell_x+1,\ell_y,\sigma}-E_{\ell_x,\ell_y,\sigma})\Big[g_+\cos\Big(\frac{\vartheta_{\ell_x+1,\ell_y}-\vartheta_{\ell_x,\ell_y}}{2}\Big)+\sigma\, g_-\cos\Big(\frac{\vartheta_{\ell_x+1,\ell_y}+\vartheta_{\ell_x,\ell_y}}{2}\Big)\Big]^2$$
$$+(\ell_x+1)\delta(\omega+E_{\ell_x+1,\ell_y,-\sigma}-E_{\ell_x,\ell_y,\sigma})\Big[g_+\sin\Big(\frac{\vartheta_{\ell_x+1,\ell_y}-\vartheta_{\ell_x,\ell_y}}{2}\Big)+\sigma\, g_-\sin\Big(\frac{\vartheta_{\ell_x+1,\ell_y}+\vartheta_{\ell_x,\ell_y}}{2}\Big)\Big]^2$$
$$+(\ell_x)\delta(\omega+E_{\ell_x-1,\ell_y,\sigma}-E_{\ell_x,\ell_y,\sigma})\Big[g_+\cos\Big(\frac{\vartheta_{\ell_x,\ell_y}-\vartheta_{\ell_x-1,\ell_y}}{2}\Big)+\sigma\, g_-\cos\Big(\frac{\vartheta_{\ell_x,\ell_y}+\vartheta_{\ell_x-1,\ell_y}}{2}\Big)\Big]^2$$
$$+(\ell_x)\delta(\omega+E_{\ell_x-1,\ell_y,-\sigma}-E_{\ell_x,\ell_y,\sigma})\Big[g_+\sin\Big(\frac{\vartheta_{\ell_x,\ell_y}-\vartheta_{\ell_x-1,\ell_y}}{2}\Big)-\sigma\, g_-\sin\Big(\frac{\vartheta_{\ell_x,\ell_y}+\vartheta_{\ell_x-1,\ell_y}}{2}\Big)\Big]^2\Big\}$$

$$J_{E_g,y}(\omega) = \tag{14}$$

$$\frac{\pi}{Z}\sum_{\ell_x,\ell_y}\sum_{\sigma} e^{-\beta E_{\ell_x,\ell_y,\sigma}}\Big\{ (\ell_y+1)\delta(\omega+E_{\ell_x,\ell_y+1,\sigma}-E_{\ell_x,\ell_y,\sigma})\Big[g_+\cos\Big(\frac{\vartheta_{\ell_x,\ell_y}-\vartheta_{\ell_x,\ell_y+1}}{2}\Big)-\sigma\, g_-\cos\Big(\frac{\vartheta_{\ell_x,\ell_y}+\vartheta_{\ell_x,\ell_y+1}}{2}\Big)\Big]^2$$
$$+(\ell_y+1)\delta(\omega+E_{\ell_x,\ell_y+1,-\sigma}-E_{\ell_x,\ell_y,\sigma})\Big[g_+\sin\Big(\frac{\vartheta_{\ell_x,\ell_y}-\vartheta_{\ell_x,\ell_y+1}}{2}\Big)+\sigma\, g_-\sin\Big(\frac{\vartheta_{\ell_x,\ell_y}+\vartheta_{\ell_x,\ell_y+1}}{2}\Big)\Big]^2$$
$$+(\ell_y)\delta(\omega+E_{\ell_x,\ell_y-1,\sigma}-E_{\ell_x,\ell_y,\sigma})\Big[g_+\cos\Big(\frac{\vartheta_{\ell_x,\ell_y-1}-\vartheta_{\ell_x,\ell_y}}{2}\Big)-\sigma\, g_-\cos\Big(\frac{\vartheta_{\ell_x,\ell_y-1}+\vartheta_{\ell_x,\ell_y}}{2}\Big)\Big]^2$$
$$+(\ell_y)\delta(\omega+E_{\ell_x,\ell_y-1,-\sigma}-E_{\ell_x,\ell_y,\sigma})\Big[g_+\sin\Big(\frac{\vartheta_{\ell_x,\ell_y-1}-\vartheta_{\ell_x,\ell_y}}{2}\Big)-\sigma\, g_-\sin\Big(\frac{\vartheta_{\ell_x,\ell_y-1}+\vartheta_{\ell_x,\ell_y}}{2}\Big)\Big]^2\Big\}$$

At low and intermediate temperatures $T \ll \omega_0$ the Boltzmann weight only allows Raman excitations from the ground state $\ell_x = \ell_y = 0$ [which is split by $\Omega \ll \omega_0$]. The above expressions then further simplify to

$$J_{E_g,x}(\omega) = \frac{\pi}{Z}\sum_{\sigma} e^{-\beta E_{0,0,\sigma}}\Big\{ \tag{15}$$
$$\delta(\omega+E_{1,0,\sigma}-E_{0,0,\sigma})\Big[g_+\cos\Big(\frac{\vartheta_{1,0}-\vartheta_{0,0}}{2}\Big)+\sigma\, g_-\cos\Big(\frac{\vartheta_{1,0}+\vartheta_{0,0}}{2}\Big)\Big]^2$$
$$+\delta(\omega+E_{1,0,-\sigma}-E_{0,0,\sigma})\Big[g_+\sin\Big(\frac{\vartheta_{1,0}-\vartheta_{0,0}}{2}\Big)+\sigma\, g_-\sin\Big(\frac{\vartheta_{1,0}+\vartheta_{0,0}}{2}\Big)\Big]^2\Big\}$$

$$J_{E_g,y}(\omega) = \frac{\pi}{Z}\sum_{\sigma} e^{-\beta E_{0,0,\sigma}}\Big\{ \tag{16}$$
$$\delta(\omega+E_{0,1,\sigma}-E_{0,0,\sigma})\Big[g_+\cos\Big(\frac{\vartheta_{0,0}-\vartheta_{0,1}}{2}\Big)-\sigma\, g_-\cos\Big(\frac{\vartheta_{0,0}+\vartheta_{0,1}}{2}\Big)\Big]^2$$
$$+\delta(\omega+E_{0,1,-\sigma}-E_{0,0,\sigma})\Big[g_+\sin\Big(\frac{\vartheta_{0,0}-\vartheta_{0,1}}{2}\Big)+\sigma\, g_-\sin\Big(\frac{\vartheta_{0,0}+\vartheta_{0,1}}{2}\Big)\Big]^2\Big\}$$

These are the function shown in the panels of Figure 4 (main text). In the schematic, the

Raman activation processes are indicated with curvy lines. The weight of each contribution is symbolically indicated through the line thickness. At low temperature $T \ll \Omega$ the sum over σ reduces to the case $|\sigma\rangle = |-\rangle$ and only the lines departing from $|0,0,-\rangle$ are contributing to the Raman signal. This results in two un-equally weighted Raman peaks for each E_g mode.

Supplementary References

---

[1] Merz, M. *et al.* Rotational symmetry breaking at the incommensurate charge-density-wave transition in $Ba(Ni,Co)_2(As,P)_2$: Possible nematic phase induced by charge/orbital fluctuations. *Phys. Rev. B* **104**, 184509 (2021).

[2] Meingast, C. *et al.*, Charge-density-wave transitions, phase diagram, soft phonon and possible electronic nematicity: a thermodynamic investigation of $BaNi_2(As,P)_2$. Preprint at https://arxiv.org/abs/2207.02294 (2022).

[3] D. R. Hamann, M. Schluter, and C. Chiang, Norm-Conserving Pseudopotentials, Phys. Rev. Lett. **43**, 1494 (1979).

[4] G.B. Bachelet, D. R. Hamann, and M. Schluter,Pseudopotentials that work: From H to P, Phys. Rev. B**6**, 4199 (1982).

[5] D. Vanderbilt, Optimally smooth norm-conserving pseudopotentials, Phys. Rev. B **32**, 8412 (1985).

[6] J.P. Perdew, K. Burke, and M. Ernzerhof, Generalized Gradient Approximation Made Simple, Phys. Rev. Lett. **77**, 3865 (1996).

[7] T. P. Devereaux and R. Hackl, *Inelastic light scattering from correlated electrons*, Rev. Mod. Phys. **79**, 175 (2007).

[8] He, M. *et al.* Dichotomy between in-plane magnetic susceptibility and resistivity anisotropies in extremely strained $BaFe_2As_2$. *Nat. Comm.* **8**, 504 (2017).

[9] Kretzschmar, F. *et al.* Critical spin fluctuations and the origin of nematic order in $Ba(Fe_{1-x}Co_x)_2As_2$. *Nat. Phys.* **12**, 560-563 (2016).
